# Antiviral responses versus virus-induced cellular shutoff: a game of thrones between influenza A virus NS1 and SARS-CoV-2 Nsp1

**DOI:** 10.3389/fcimb.2024.1357866

**Published:** 2024-02-05

**Authors:** Ahmed Magdy Khalil, Aitor Nogales, Luis Martínez-Sobrido, Ahmed Mostafa

**Affiliations:** ^1^ Disease Intervention & Prevention and Host Pathogen Interactions Programs, Texas Biomedical Research Institute, San Antonio, TX, United States; ^2^ Department of Zoonotic Diseases, Faculty of Veterinary Medicine, Zagazig University, Zagazig, Egypt; ^3^ Center for Animal Health Research, CISA-INIA-CSIC, Madrid, Spain; ^4^ Center of Scientific Excellence for Influenza Viruses, National Research Centre, Giza, Egypt

**Keywords:** influenza A virus, SARS-CoV-2, non-structural protein 1, NS1, Nsp1, innate immunity, live attenuated vaccines, antivirals

## Abstract

Following virus recognition of host cell receptors and viral particle/genome internalization, viruses replicate in the host via hijacking essential host cell machinery components to evade the provoked antiviral innate immunity against the invading pathogen. Respiratory viral infections are usually acute with the ability to activate pattern recognition receptors (PRRs) in/on host cells, resulting in the production and release of interferons (IFNs), proinflammatory cytokines, chemokines, and IFN-stimulated genes (ISGs) to reduce virus fitness and mitigate infection. Nevertheless, the game between viruses and the host is a complicated and dynamic process, in which they restrict each other via specific factors to maintain their own advantages and win this game. The primary role of the non-structural protein 1 (NS1 and Nsp1) of influenza A viruses (IAV) and the pandemic severe acute respiratory syndrome coronavirus 2 (SARS-CoV-2), respectively, is to control antiviral host-induced innate immune responses. This review provides a comprehensive overview of the genesis, spatial structure, viral and cellular interactors, and the mechanisms underlying the unique biological functions of IAV NS1 and SARS-CoV-2 Nsp1 in infected host cells. We also highlight the role of both non-structural proteins in modulating viral replication and pathogenicity. Eventually, and because of their important role during viral infection, we also describe their promising potential as targets for antiviral therapy and the development of live attenuated vaccines (LAV). Conclusively, both IAV NS1 and SARS-CoV-2 Nsp1 play an important role in virus–host interactions, viral replication, and pathogenesis, and pave the way to develop novel prophylactic and/or therapeutic interventions for the treatment of these important human respiratory viral pathogens.

## 1 Introduction

Respiratory viruses (RVs) are annually reported as the most communicable causative agent of human hospitalization and the most predominant etiological agents of acute respiratory distress syndrome (ARDS), leading to significant impacts on morbidity and mortality globally (Shah and Wunderink, 2017). RVs that commonly circulate annually in all continents as endemic or epidemic agents include mainly influenza viruses, low pathogenic coronaviruses, respiratory syncytial virus, metapneumovirus, parainfluenza viruses, rhinoviruses, bocaviruses and respiratory adenoviruses. Influenza viruses led to at least three pandemics during the last century and one pandemic at the beginning of this century (Mostafa et al., 2018). Remarkably, the influenza pandemic of 1918/1919 or “Spanish flu”, caused by influenza A/H1N1 virus, and the coronavirus disease 2019 (COVID-19) pandemic of 2019, caused by severe acute respiratory syndrome coronavirus 2 (SARS-CoV-2), are considered the most tragic communicable disease that have been associated with enormous health and economic burdens in humans (James, 2021; Morens et al., 2021).

Influenza viruses are categorized into four types: influenza A viruses (IAV), influenza B viruses (IBV), influenza C viruses (ICV), and influenza D viruses (IDV) (Mostafa et al., 2018). IAV contains eight negative sense (-) viral (v)RNA segmented genome (∼13.5 kilobases (kb)) which encode, at least, 10 viral proteins: the polymerase basic protein 2 (PB2, segment 1), the polymerase basic protein 1 (PB1, segment 2), the polymerase acidic protein (PA, segment 3), hemagglutinin (HA, segment 4), nucleoprotein (NP, segment 5), neuraminidase (NA, segment 6), matrix proteins (M1 and its splicing product M2, segment 7) and the non-structural protein (NS1) and its splicing product, the nuclear export protein (NEP) (segment 8) (Mostafa et al., 2018). IAV are classified according to their surface glycoproteins (HA and NA) into 18 and 11 distinct subtypes, respectively (Bahgat et al., 2009; Mostafa et al., 2018; Naguib et al., 2019).

Coronaviruses (CoV) are divided also into four genera, namely alpha, beta, gamma, and delta that infect multiple animal species, including humans (Mostafa et al., 2020; Al-Karmalawy et al., 2021). Only 7 viruses that belong to the alpha and beta CoV genera could escape species barrier to infect humans, including four low pathogenic human coronaviruses (hCoV), namely 229E (alpha CoV), NL63 (alpha CoV), OC43 (beta CoV) and HKU1 (beta CoV); and three highly pathogenic hCoV: Middle East Respiratory Syndrome (MERS)-CoV (beta CoV), Severe Acute Respiratory Syndrome (SARS)-CoV (beta CoV), and SARS-CoV-2 (beta CoV) (Mostafa et al., 2020; Al-Karmalawy et al., 2021). The genome of hCoV is comprised of a single-stranded positive-sense (+) RNA genome which is among the largest viral RNAs known to date (∼30 kb) (Grellet et al., 2022).

During IAV or hCoV attachment and subsequent entry, host cells recognize the invading viral particle via pattern recognition receptors (PRRs) to initiate the secretion of virus-induced antiviral cytokines, chemokines, interferon (IFN), and IFN-stimulated genes (ISGs) (Wiwie et al., 2019; Liu et al., 2022a). Following host cell infection, IAV and hCoV utilize several strategies to hamper IFN expression (Hale et al., 2008b; Evseev and Magor, 2021). Most of these strategies are depending mainly on two non-structural viral protein(s), specifically IAV NS1 and hCoV non-structural protein 1 (Nsp1), that hijack, suppress, or escape the host cell innate immunity antiviral responses via several molecular strategies, including antagonism of IFN production (Kanrai et al., 2016; Petersen et al., 2018; Khorramdelazad et al., 2021; Kumar et al., 2021a; Blake et al., 2023).

**Figure 1:**
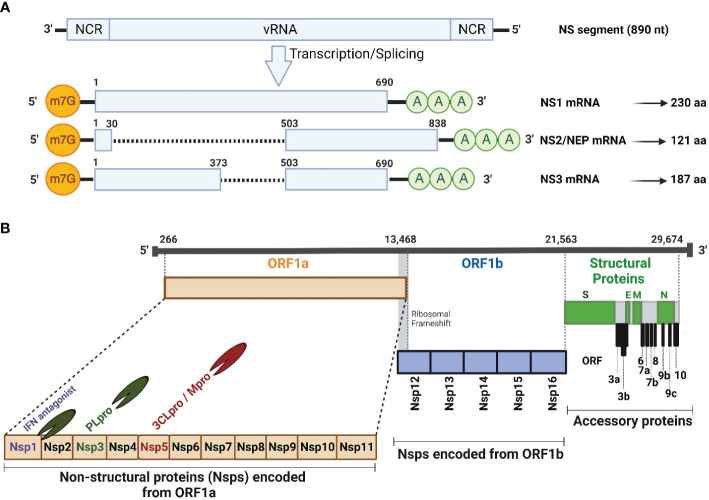
Schematic illustration of IAV NS segment and SARS-CoV-2 genome organization. **(A)** Schematic representation of the unspliced and spliced mRNA forms transcribed from the IAV NS vRNA segment. The unspliced mRNA transcript encodes for the NS1 protein while the spliced mRNA transcripts encode the NS2 or NEP and NS3 protein which is typically the NS1 mRNA transcript with an internal deletion. **(B)** SARS-CoV-2 vRNA genome organization and viral structural (S, E, M and N; green), accessory (ORF 3a, 3b, 6, 7a, 7b, 8, 9b, 9c and 10; gray), and non-structural (Nsp1-16) proteins encoded from ORF1a (orange) and ORF1b (light blue). HCoV Nsp1 is expressed from the N-terminal of ORF1a. NCR, Non-coding region; ORF, Open reading frame; m7G, 7-methylguanosine (m7G) cap structure; AAA, poly **(A)** tail; Nsp, Non-structural protein; PLpro, papain-like protease; 3CLPro, 3-chymotrypsin-like protease; Mpro, main protease. Figure was created with BioRender.com.

IAV NS1 is a multifunctional protein, encoded by the 8^th^ or NS viral RNA (vRNA) segment and is translated from the unspliced viral mRNA (**Figure 1A**). Based on sequence homology, IAV NS1 is generally divided into at least 2 distinct alleles. NS1 Allele “A” is most often found in all IAV isolates from mammals and some avian strains, whereas NS1 allele “B” is restricted to some avian IAV (Evseev and Magor, 2021). In addition, a further allele represents the NS1 proteins of IAV-like H17 and H18 bat isolates, which shares only ∼50% sequence identity with other IAV NS1 proteins (Turkington Hannah et al., 2018). Nevertheless, an infectious recombinant influenza A/H1N1 expressing the NS1 but not the NEP of H17N10 could be rescued (Zhao et al., 2016), demonstrating potential genetic compatibility of NS1 from IAV-like bat viruses and other IAV. IAV NS1 has a typical length of 230 amino acids (aa), and an approximate molecular weight of 26 kDa (Ji et al., 2021). However, NS1 varies in length (202-237 aa) among different IAV subtypes and strains due to premature stop codons, or contrarily, the suppression of the most prevailing stop codon at nucleotide 688–690 that produce NS1 proteins with truncations or insertions at the disordered C-terminal tail, respectively (Hale et al., 2008b; Petersen et al., 2018; Trigueiro-Louro et al., 2019; Blake et al., 2023). Even though IAV NS1 protein has no apparent structural function in the viral particle, it contributes efficiently to viral replication and pathogenicity by suppressing IFN production and the activity of many ISGs (Petersen et al., 2018; Blake et al., 2023). IAV NS1 also contributes to the shut-down of host gene expression by preventing the cellular splicing and polyadenylation machineries of cellular mRNA and blocking the nuclear export of polyadenylated mature mRNAs (Nemeroff et al., 1998; Ayllon and Garcia-Sastre, 2015; Evseev and Magor, 2021). These activities of IAV NS1 on cellular mRNA transcripts lead to the aggregation of premature mRNAs in the nucleus to be used as a template for cap-snatching to initiate the synthesis of viral mRNA transcripts (Nemeroff et al., 1998).

The spliced IAV NS mRNA, encoding NEP (121 aa, 14.4 kDa) (Golovko et al., 2017), harbors the same initiation codon (AUG) and the sequence of the first 10 aa of IAV NS1 protein (**Figure 1A**). Intriguingly, IAV NEP is found in association with the matrix 1 (M1) protein within the virion and is implicated, together with the chromosomal maintenance 1 (CRM1) nuclear export protein, in mediating the nuclear export of viral ribonucleoprotein (vRNP) complexes in the late stage of the viral replication cycle (Dou et al., 2018). This emphasizes that NEP is packaged, together with other structural viral proteins, into the newly generated viral particles. In addition to its ability to regulate the nuclear export of vRNPs, NEP has several influential functions during IAV replication. It has been reported to contribute to the viral budding process through interaction with a cellular ATPase (Gorai et al., 2012), and it can control the accumulation of vRNA species leading to transition from early transcription to the genome replication and vRNP production (Paterson and Fodor, 2012). Recently, Zhang et al. has elucidated the role of NS1 in regulating viral transcription (vRNA→mRNA) versus viral replication (vRNA→cRNA→vRNA) in coordination with the NS2 protein (Zhang et al., 2023). The early-expressed NS1 protein could promote viral mRNA synthesis, while the late-expressed NS2 protein inhibits mRNA synthesis and boost the vRNA synthesis in accordance with the dynamic changes in mRNA/vRNA during virus replication cycle, confirming the regulatory role of the viral NS1 and NS2 proteins during IAV infection (Zhang et al., 2023).

On the same hand, IAV non-structural 3 (NS3) protein was naturally found in a minority of IAV strains that lack NS1 protein (Selman et al., 2012) (**Figure 1A**). IAV NS3 protein (187 aa, 20 kDa) is encoded by a spliced mRNA transcript, which is like that of NS1, but with an internal gap. IAV NS3 protein has a biological function like that of IAV NS1, nevertheless, with different nuclear and cellular localization (Selman et al., 2012; Hao et al., 2020).

The hCoV genome is flanked by conserved 5’ and 3’ untranslated regions (UTR) that form complex secondary structures that play important roles in initializing viral genome transcription and replication (Mohammadi-Dehcheshmeh et al., 2021) (**Figure 1B**). The coding genome of hCoV starts commonly with two large (2/3 of viral genome) and overlapping 5′-terminal open reading frames (ORF), namely ORF1a and ORF1b. Both ORF encode large polyproteins that subsequently are cleaved into 16 non-structural proteins (Nsp, Nsp1-Nsp16) with important roles in viral replication, pathogenicity, and transmission (Redondo et al., 2021; Yadav et al., 2021). Due to their important contribution in viral replication, the 16 Nsp are among the early expressed viral proteins in hCoV-infected cells following viral genome release into the host cell cytosol (Naqvi et al., 2020). Interestingly, SARS-CoV-2 encodes two proteases: papain-like protease (PL_pro_) and 3-chymotrypsin-like protease or main protease (3CL_Pro_ or M_pro_), which are parts of Nsp3 and Nsp5, respectively (Fu et al., 2021; Cheng et al., 2023) (**Figure 1B**). The PL_pro_ recognizes and hydrolyzes the tetrapeptide LXGG motif between the viral Nsp1/2, Nsp2/3, and Nsp3/4 (Han et al., 2005), to release individual Nsp1, Nsp2, and Nsp3 proteins. The other 13 Nsp are processed further by the 3CL or M_pro_ protease (Han et al., 2005). Interestingly, hCoV utilizes the Nsp1 as a host shutoff factor to counteract host antiviral activities via suppressing host gene expression and the host immune response, including the production of IFN and ISGs that control virus replication (Kumar et al., 2021a).

The remaining hCoV genome encodes mainly the virus structural proteins including spike (S), envelope (E), membrane (M), and nucleocapsid (N), and some accessory ORF proteins that are hCoV-strain specific. For instance, SARS-CoV encodes ORF3a, ORF3b, ORF6, ORF7a, ORF7b, ORF8a, ORF8b, and ORF9, however SARS-CoV-2 encodes ORF3a, ORF3b, ORF6, ORF7a, ORF7b, ORF8, ORF9b, ORF9c and ORF10; while four accessory ORF proteins were documented in the case of MERS-CoV, including ORF3, ORF4a, ORF4b, and ORF5 (Hurtado-Tamayo et al., 2023).

## 2 IAV NS1 and SARS-CoV-2 Nsp1 structures

### 2.1 IAV NS1 structure

**Figure 2:**
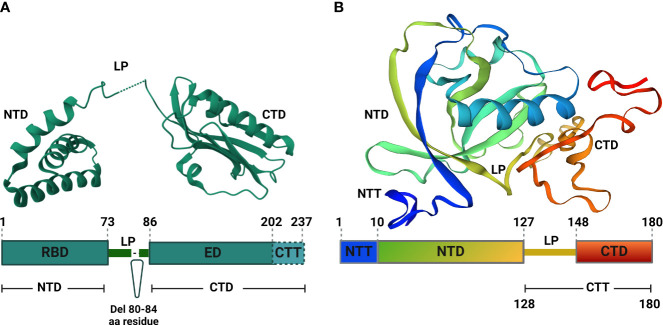
Structural analyses of IAV NS1 and SARS-CoV-2 Nsp1. **(A)** Functional motifs of IAV NS1 include the RNA-binding domain (RBD) or N-terminal domain (NTD), followed by a short linker peptide (LP), the C-terminal domain (CTD) or effector domain (ED), and the strain specific C-terminal tail (CTT). Amino acid (aa) residues are indicated by numbers in the primary structure of IAV NS1 (bottom). IAV NS1 monomer adapted from the X-ray structure of H5N1 NS1 (PDB ID: 3F5T) (Bornholdt and Prasad, 2008), showing the NTD, LP with a 5 aa deletion (residues 80-84), predominant in IAV H5N1 NS1 since 2003 (Li et al., 2014), and the CTD (top). **(B)** SARS-CoV-2 Nsp1 consists of a short N-terminal tail (NTT), an N-terminal domain (NTD), and a C-terminal tail (CTT) that contains a linker peptide (LP) and the C-terminal domain (CTD). The aa residues are indicated by numbers in the primary structure of SARS-CoV-2 Nsp1 (bottom). SARS-CoV-2 Nsp1 monomer adapted from the X-ray structure (PDB: 8AOU) is shown on top (Wang et al., 2023). Figure was assembled with BioRender.com.

IAV NS1 protein is involved in the interactions with multiple host cellular factors, especially those involved in antiviral host responses (Kanrai et al., 2016). Based on the three-dimensional structure, IAV NS1 is composed of two functional domains: the N-terminal (aa residues 1-73) domain (NTD), also known as RNA-binding domain (RBD); and the C-terminal (aa residues 86-207) domain (CTD), also known as effector domain (ED) that are linked together by a flexible short (aa residues 74-85) linker peptide (LP) (**Figure 2A**) (Qian et al., 1994; Carrillo et al., 2014).

The spatial structure of IAV NS1 RBD exists as a symmetrical compact homodimer with three α-helices connected by short loops in each RBD monomer (**Figure 2A**) (Bornholdt and Prasad, 2008). The three α-helices of the RBD monomer homodimerize to constitute the antiparallel six α-helices bundle (Bornholdt and Prasad, 2008; Hale et al., 2008a; Hale et al., 2008b). Likewise, the IAV NS1 ED also exists as homodimer to primarily mediate interactions with various cellular proteins and stabilizes RNA-binding functions of the RBD (**Figure 2A**) (Hale et al., 2008b). Each IAV NS1 ED monomer is formed of three α-helices and seven antiparallel β-strands, which homodimerize independently (**Figure 2A**) (Kerry et al., 2011).

Dimerization is an absolute prerequisite for the RNA-binding activity because the dimerization of identical helices of IAV NS1 RBD monomer result in antiparallel track with conserved basic and hydrophilic aa residues (T5, P31, N34, R35, R38, K41, G45, R46 and T49) (Melén et al., 2007). This formed track contribute towards double-stranded (ds)RNA-binding via interaction with the polyphosphate backbone of dsRNA, especially at positions R38 and K41, to sit and stabilize the interaction with the dsRNAs (Melén et al., 2007). Arginine (R) to alanine (A) or lysine (K) to A mutations at position 38 (R38A) and 41 (K41A) of IAV NS1 destroy its dsRNA binding activity (Min and Krug, 2006). Contrary to the RBD dimers, IAV NS1 ED dimers are weak (Kerry et al., 2011). This weak dimerization is important for the regulation of IAV NS1 activities because it allows flexible orientation of the two ED monomers, allowing reversible interactions with itself and/or with other cellular host factors (Kerry et al., 2011; Hale, 2014).

The IAV NS1 LP between the RBD and ED is mostly made of 11 aa residues (74-85) (Hale et al., 2008b). The majority of IAV H5N1 isolates circulating after 2003 displayed a 5 aa deletion from residues 80 to 84 in the LP (**Figure 2A**) (Blake et al., 2023). Despite that the exact role of the LP in not fully understood, studies showed that it is crucial for the distinct properties of IAV NS1 because it controls the large conformational changes required to switch from the ‘helix-open’ to ‘helix-closed’ state and vice versa (Kerry et al., 2011; Hale, 2014). Consequently, variation in the length and/or sequence of the IAV NS1 LP could alter the viral phenotype, including viral replication and pathogenicity (Li et al., 2011; Kanrai et al., 2016; Evseev and Magor, 2021). Beside the ED, the CTD comprises also an intrinsically disordered C-terminal tail (aa residues 202-230) that is highly convertible in length due to truncations (up to 28 aa) or extensions (7 aa, human IAV), arising through viral evolution (Hale et al., 2008a). This CTD contributes to strain-specific functions via interaction with host cellular proteins (Evseev and Magor, 2021).

### 2.2 SARS-CoV-2 Nsp1 structure

SARS-CoV-2 Nsp1 comprises a short N-terminal tail (NTT, aa residues 1-9), an N-terminal domain (NTD, aa residues 10-127), an α/β fold formed by a six-stranded, capped β-barrel-like globular domain, and a long C-terminal tail (CTT, aa residues 128-180). The CTT features a flexible 20 aa linker peptide region (LP, aa residues 128-147) and a short C-terminal domain made of two short alpha helices (CTD, aa residues 148-180) (**Figure 2B**) (Schubert et al., 2020a; Clark et al., 2021; Wang et al., 2023). Structurally, SARS-CoV-2 Nsp1 is a monomer in solution (Wang et al., 2023), and executes its function(s) in harmony between its fundamental regions, the NTD and the CTD (Thoms et al., 2020). For instance, the CTD colocalizes mainly with the ribosomes to inhibit host gene translation (Zhao et al., 2021) and this function is significantly weakened in the absence of the NTD that stabilize the binding of CTD to the ribosomes and act as a non-specific blocker of the mRNA channel, inhibiting host mRNA translation (Zhao et al., 2021).

## 3 IAV NS1 and SARS-CoV-2 Nsp1 post-translational modifications and cellular localization

### 3.1 Post-translational modifications and localization of IAV NS1 in infected cells

**Figure 3:**
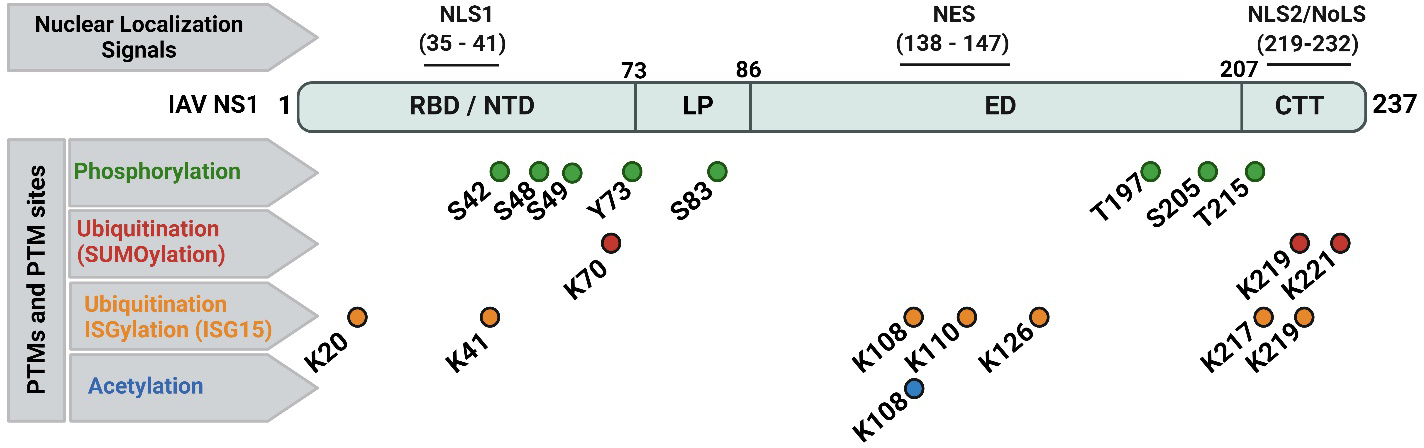
Nuclear localization signals (NLS), nuclear export signal (NES), and documented post-translational modifications (PTMs) of IAV NS1 protein. Typical IAV NS1 proteins contain two nuclear localization signals (NLS1 and NLS2). The NLS2 is overlapping with a nucleolar localization signal (NoLS) forming the nuclear/nucleolar localization signal (NLS2/NoLS). In addition, IAV NS1 also contains a NES. IAV NS1 protein is usually subjected to crucial PTM in infected cells, including phosphorylation (green), ubiquitination via SUMOylation (red) and ISGylation (orange), and acetylation (blue). The PTMs are indicated according to their position in IAV NS1. Figure was created with BioRender.com.

IAV NS1 is one of the most abundant proteins produced during viral replication in infected cells (Nemeroff et al., 1998; Terrier et al., 2012). IAV NS1 is subjected to post-translational modifications (PTMs) (**Figure 3**) including phosphorylation at threonine (T) 49 and T215, and at serine (S) 42 and S48 aa residues (Hsiang et al., 2012; Kathum et al., 2016). Phosphorylation of IAV NS1 at S42, but not S48 or T215, does play a crucial role in viral replication (Hsiang et al., 2012). Phosphorylation at T49 (located in the RBD area) could influence binding of IAV NS1 to dsRNA, tripartite motif-containing protein 25 (TRIM25) and retinoic acid-inducible gene I (RIG-I), thereby affecting its IFN antagonistic activity (Kathum et al., 2016). Likely, lack of phosphorylation of the pandemic 2009 IAV H1N1 (H1N1pdm09) NS1 at tyrosine (Y) residue 73 and S83, due to mutations Y73F and S83A, respectively, has been shown to affect the ability of IAV NS1 to inhibit host antiviral responses, partly mediated via RIG-I pathway (Nacken et al., 2014; Cheng et al., 2019). Phosphorylation of IAV pandemic (p)H1N1 NS1 at residue S205 is mediated by the cellular kinase CK2, resulting in efficient interaction of the H1N1pdm09 NS1 with the human host restriction factor “DExD-Box Helicase 21” (DDX21) to further support NS1-induced enhancement of viral polymerase activity (Patil et al., 2021).

Two types of ubiquitination have been reported in IAV NS1 (**Figure 3**): (a) SUMOylation (SUMO1-linkage, a PTM of K aa residues on a protein substrate by a small ubiquitin-like modifier (SUMO) protein conjugation); and (b) ISGylation (ISG15-linkage, a PTM induced by IFN stimulation of ISG15). Despite the need of the IFN blocking of IAV NS1 for optimal SUMOylation, SUMOylation of K at IAV NS1 aa residues 70 and 219 does not affect its stability or cellular localization but can affect its oligomerization and therefore NS1’s functions (Santos et al., 2013). In addition, specific SUMOylation sites at residues K219 and K221 were characterized in the NS1 of avian H5N1 IAV to be essential to maintain NS1 stability and ability to antagonize host protein expression (Xu et al., 2011). On the same hand, the ISG15-conjugated K in IAV NS1 protein were identified by mass spectrometry at aa residues 20, 41, 67, 70, 110, 126, 196, and 219 (Zhao et al., 2010). Moreover, E3 ubiquitin-protein ligase (HERC5)-mediated ISGylation of K residues at 7 aa positions including 20, 41, 217, 219 in NTD or CTD of IAV NS1; and 108, 110, and 126 in the middle part of IAV-NS1 could hinder NS1 dimerization and hence inhibit relevant antiviral antagonistic interactions and potentiate IFN antiviral responses (Tang et al., 2010). The ISG15-linkage to K41 can also block the interaction of IAV NS1 RBD domain with the cellular importin-α, thereby impairing IAV NS1 function(s), and, therefore, affecting viral replication (Zhao et al., 2010).

The third important PTM of IAV NS1 is acetylation (**Figure 3**). Acetylation of IAV NS1 at K108 plays an important role in viral replication and virulence (Ma et al., 2020). The K108R deacetylation in IAV NS1 has been associated with a reduced ability of NS1 to inhibit IFN, attenuating IAV H1N1 replication and virulence in mice, while the IAV H1N1 expressing constant acetylation-mimic mutation (K108Q) showed comparable replication and virulence to the wild-type (WT) IAV H1N1 containing the NS1 K108 acetylation site (Ma et al., 2020).

IAV NS1 contains a conserved nuclear localization sequence (NLS1; aa residues 35-41) and a nuclear export sequence (NES; aa residues 138-147) (**Figure 3**). A nuclear/nucleolar localization signal (NLS2/NoLS; aa residues 219-232) is also present in some human IAV strains with elongated CTD (237 aa). At early stage of the replication cycle, IAV NS1 is mainly localized to the nucleus. However, at later times after infection, IAV NS1 is found in the cytoplasm of infected cells (Shaw and Palese, 2013). IAV NS1 highly conserved NLS1 is composed of basic aa residues (R at positions 35 and 38; and K at position 41) in the helix α-2 of the RBD and contributes to its nuclear import by interaction with the cellular importin-α (Greenspan et al., 1988; Melén et al., 2007). At the CTT disordered tail, some IAV NS1 also contain the NLS2, which includes the basic aa residues K219 and R220, R231 and R232; and a functional NoLS, which includes additional aa residues R224 and R229 (Melén et al., 2007; Melen et al., 2012). Conversely, IAV NES, located in the ED and composed of the aa residues from positions 138 to 147, contributes to the cytoplasmic redistribution of NS1 (Li et al., 1998). Functional NES requires aa residues L144 and L146, and can be altered by aa changes at residues 148–161 which lie in its vicinity (Li et al., 1998; Mok et al., 2017). Therefore, deletions or mutations in the NES domain led to either a nuclear retention or impaired localization of IAV NS1 (Li et al., 1998). For instance, the nuclear NS1 localization was not detected following A549 cell infection with influenza A/WSN/1933 (WSN; H1N1) expressing mutated NS1 protein, namely WSN/NS1-R148A/E152A/E153A. The viral replication and transcription efficacies of both WSN/NS1-R148A/E152A/E153A mutant virus and WSN/ΔNS1 virus (missing NS1 protein) were significantly decreased compared to the WSN virus, attenuating these viruses in their response to the host immunity (Mok et al., 2017).

### 3.2 PTMs and cellular localization of SARS-CoV-2 Nsp1 during viral infection

Unlike IAV that replicate within the nucleus of the host infected cell, SARS-CoV-2 lacks a nuclear replication step and replicates only in the cytoplasm of infected cells (Cohen et al., 2011; Chen et al., 2022). Nevertheless, one of the known mechanisms by which SARS-CoV-2 Nsp1 inhibit host gene expression/translation occurs via hindering the nuclear transport complex NXF1/NXT1 to mediate the nuclear export of host mRNA from the nucleus to the cytoplasm of infected cells for translation (Zhang et al., 2021; Devarkar et al., 2023). Although recent studies demonstrated that the SARS-CoV-2 Nsp1 is localized mainly in the cytoplasm of infected cells, a small portion colocalizes with the components of the nuclear pore complexes (NPCs) at the nuclear envelope, emphasizing that Nsp1 can indirectly displace NXF1/NXT1 complex from its NPC interactors (Zhang et al., 2021; Devarkar et al., 2023). This finding confirm that SARS-CoV-2 Nsp1 can achieve this function at the interface between cytoplasmic and nuclear compartments of the host infected cell without localization into the nucleus. Nothing is known about potential nuclear localization (NLS) or export (NES) signals in SARS-CoV-2 Nsp1. Likewise, little is known about PTMs of structural and non-structural proteins of hCoV, including SARS-CoV-2. To date, no PTMs have been described for SARS-CoV-2 Nsp1 (Fung and Liu, 2018; Cheng et al., 2023).

## 4 IAV NS1 and SARS-CoV-2 Nsp1 interactions with viral and cellular proteins

### 4.1 IAV NS1 and its interaction with viral and cellular proteins

**Figure 4:**
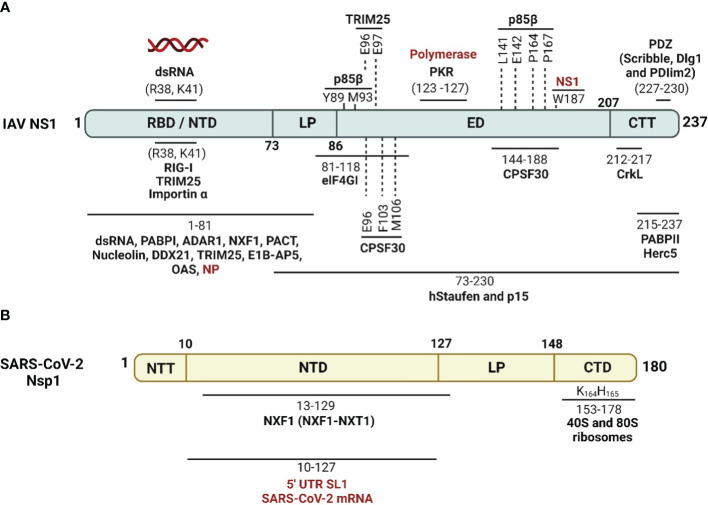
Schematic representation of cellular/viral interactors of IAV NS1 and SARS-CoV-2 Nsp1. **(A)** IAV NS1 interacts with four groups of proteins, which are involved in the maturation and nucleo-cytoplasmic export of cellular mRNAs (CPSF30, PABPII, nucleolin, NXF1/TAP and several other proteins of the nuclear export machinery), proteins involved in mRNA transport and translation (hstaufen, eIF4GI and PABPI), proteins involved in the IFN antiviral response and signaling cascades (RIG-I, TRIM-25, PKR, PACT, p85-b subunit of PI3K and PDZ-containing proteins), and eventually viral proteins including NP and viral polymerase components (highlighted in red) (Knipe and Howley, 2013). **(B)** SARS-CoV-2 Nsp1 shuts off innate immune responses via indirect or direct interaction of its NTD with fundamental cellular proteins, including NXF1, and interaction of its CTD with the 40S and 80S ribosome subunits. SARS-CoV-2 Nsp1 NTD can also selectively mediate the translation of viral mRNA via interaction with the stem-loop 1 (SL1) region of the SARS-CoV-2 5’ untranslated region (UTR, highlighted in red). Figure was created with BioRender.com.

IAV NS1 protein interacts with a high number of cellular proteins as well as some viral proteins to modulate innate immune responses (Hale et al., 2008b; Nogales et al., 2018b) (**Figure 4A**). According to their biological activities, these proteins are divided into four groups as reviewed in (Marc, 2014): (1) The nuclear and RNA metabolism proteins, involved in the maturation and nucleo-cytoplasmic export of cellular mRNAs, including the cleavage and polyadenylation specificity factor (CPSF30), a 30 kDa subunit of the cleavage and polyadenylation specificity factor, poly (A)-binding protein II (PABPII), nucleolin, NXF1/TAP and several proteins of the nuclear export machinery (e.g. Rae1 and E1B-AP5). (2) Cytosolic/ribosomal proteins, involved in mRNA transport and translation, including staufen, elongation initiation factor 4GI (eIF4GI), and poly (A)-binding protein I (PABPI). (3) IFN proteins, involved in IFN production and IFN signaling pathways, including RIG-I, TRIM25, protein kinase RNA-activated (PKR), PKR activator (PACT), the regulatory subunit p85β of phosphatidylinositol 3-kinase (PI3K), and PDZ-containing proteins. (4) Viral proteins, including NS1 interaction with NP in a complex with CPSF30 and the viral polymerases.

These cellular and viral proteins interact with different domains and in different ways with IAV NS1. The majority of these IAV NS1 partners target the ED. However, some others prefer to interact with the IAV NS1 RBD directly like importin-α (Melén et al., 2007), or indirectly via the bound dsRNA. The binding of IAV NS1 to specific dsRNA may prevent or attract dsRNA-binding proteins of the same targets (Aramini et al., 2011). Consequently, IAV NS1 could impair the function of these host cell proteins or compete with them for their target dsRNA. The polymorphism of IAV NS1 proteins triggers variable interactions with its partners (Shapira et al., 2009; Rajsbaum et al., 2012; de Chassey et al., 2013). Moreover, the highly conserved aa residue W187 at the ED mediates homotypic NS1 interaction (Ayllon et al., 2012).

On the same hand, NS1 of different IAV subtypes shows differential interaction patterns with the cellular factors which ultimately dictates the pathogenicity of these IAV subtypes (Turkington Hannah et al., 2018; Monteagudo et al., 2019). Recombinant IAV expressing human influenza A/H1N1 NS1 or A/H3N2 NS1 induced higher levels of type I and III interferons (IFN-I/III) than those expressing avian influenza A/H5N1 NS1, A/H7N9 NS1 and A/H7N2 NS1 in dendritic cells (DCs), suggesting that avian NS1 proteins have increased capability to antagonize IFN-I/III production (Monteagudo et al., 2019). Compared to classical IAV NS1, bat IAV NS1 shows inability to bind the host p85β or activate PI3K signaling (Turkington Hannah et al., 2018). Collectively, these findings confirm that IAV NS1 are interacting with their cellular interactors in a strain-specific manner.

### 4.2 SARS-CoV-2 Nsp1 interaction with viral and cellular proteins

SARS-CoV-2 Nsp1 interferes with host IFN responses via interaction with distinct host proteins to inhibit mRNA translation, trigger host mRNA cleavage and reduce cytokines and chemokines (Zhang et al., 2021) (**Figure 4B**). A recent study showed that SARS-CoV-2 Nsp1 is the second major viral protein to interact with peptides from human proteins (Mihalič et al., 2023). Nevertheless, the mechanism underlying the impact of cellular proteins’ interaction with SARS-CoV-2 Nsp1 in infected cells are not fully identified (Mihalič et al., 2023).

In the cytoplasm, SARS-CoV-2 Nsp1 colocalizes and binds to the human 40S ribosomal subunit in the ribosomal complex, including the 43S pre-initiation complex and the non-translating 80S ribosome (Schubert et al., 2020a). Subsequently, SARS-CoV-2 Nsp1 insert its CTD into the mRNA entry channel to physically block it and cease mRNA translation (Schubert et al., 2020a). This process involves interactions of SARS-CoV-2 Nsp1 C-terminal α-helix (aa residues 153–160 and interactive aa residues Y154 and F157) and the essential ribosomal “uS3 and uS5” (Schubert et al., 2020a) that play a role as RNA helicase and accommodates the incoming cellular mRNA for proper codon reading in the ribosome A site, respectively (Kurkcuoglu et al., 2008). The second α-helix of SARS-CoV-2 Nsp1 (aa residues 166–178), interacts with the phosphate backbone of helix h18 of the 18S rRNA via the two conserved R residues at aa positions 171 and 175 (Schubert et al., 2020a). The conserved KH motif at aa residues 164 and 165 of SARS-CoV-2 Nsp1 CTD was shown to be crucial for ribosome binding and translation inhibition (Thoms et al., 2020).

SARS-CoV-2 Nsp1 can selectively impair cellular mRNA translation but not viral mRNA by proper coordination of its NTD and CTD interaction with the conserved 5′ UTR stem-loop 1 (SL1) of SARS-CoV-2 mRNA (Schubert et al., 2023). The 5’ UTR of SARS-CoV-2 mRNA binding to Nsp1 NTD could dissolve the Nsp1 CTD binding to the 40S ribosomal subunit in the ribosomal complex, giving preference to viral mRNA translation and accumulation of cellular mRNA (Schubert et al., 2023). This activates the endonucleolytic cleavage and subsequent degradation of accumulated mRNAs that reduces the cellular protein levels of essential innate immunity elements, including IFNs, RIG-I, and ISG15 (Thoms et al., 2020). Additionally, SARS-CoV-2 Nsp1 disrupts the binding of the subunit “j” of the multi-subunit initiation factor (eIF3j) to the 40S ribosomal subunit (Yuan et al., 2020). SARS-CoV-2 Nsp1 also antagonizes IFN via preventing phosphorylation of the IFN regulatory factor (IRF)3, possibly through translational shutoff that depletes the required cellular factors involved in IRF3 phosphorylation, including tyrosine kinase (TYK)2 and signal transducer and activator of transcription (STAT)2 (Fiege et al., 2021; Kumar et al., 2021a).

Similar to IAV NS1 that can inhibit host mRNA export via direct interaction with the mRNA export receptor heterodimer NXF1-NXT1 and nucleoporins (Satterly et al., 2007), and also with the mRNA nuclear export factor Rae1 and E1B-AP5 [60], SARS-CoV-2 Nsp1 impair the recruitment of NXF1 to the mRNA via interfering with export factors (e.g. THO complex and Aly/REF (THOC4)) and NXF1 docking to the nuclear pore complex, hindering mRNA export to the cytoplasm for translation (Zhang et al., 2021). The most important and well characterized cellular and viral proteins involved in interaction with IAV NS1 and SARS-CoV-2 Nsp1 are shown in **Tables 1**, **2**, respectively.

**Table 1:** Viral and host interactors of IAV NS1.

Target	Type	Mechanism	Interacting motifs	Citation(s)
RIG-I pathway	Cellular	- Hinders RIG-I activation directly through competitive interaction with dsRNAs. - Indirectly hinders RIG-I activation by blocking TRIM-25- and RIPLET-mediated ubiquitination of RIG-I and hence impairing RIG-I signaling pathways. - Suppresses PACT/RIG-I-mediated activation of IFN.	38-41 1-73	(Klemm et al., 2018; Zhu et al., 2022)
PKR	Cellular	- Directly binds and blocks the activation of PKR, resulting in impaired phosphorylation and subsequent nuclear translocation of IFN transcription factors, including nuclear factor kappa-light-chain-enhancer of activated B cells (NF-κB) and IRF3.	123-127	(Klemm et al., 2018; Zhu et al., 2022)
p85β/PI3K	Cellular	- Directly binds to p85β, a regulatory subunit of PI3K and stimulates the lipid kinase activity of p85β-associated p110, activating PI3K signaling that is important for efficient IAV replication. - Independently of IFN, IAV NS1-mediated activation of PI3K has been shown to support anti-apoptotic pathways.	89, 164, 167	(Hale et al., 2006; Klemm et al., 2018; Zhu et al., 2022)
OAS/RNase L	Cellular	- Sequesters dsRNA required for activating the 2`-5` oligo-adenylate synthetase (OAS)/RNase L. The OAS/RNase L system is an innate immunity pathway that responds to dsRNA as a pathogen-associated molecular pattern molecule (PAMP) to induce degradation of viral and cellular RNAs and thereby block viral infections. - Sequesters dsRNA, PKR, and OAS/RNase L, required to induce IFN and to trigger apoptosis following IAV infection. Sequestration of these elements and inhibition through interaction with NS1 interferes with IAV-induced apoptosis.	38, 41	(Klemm et al., 2018; Zhu et al., 2022)
CPSF30	Cellular	- Shuts off the 3′-end processing and translation of the cellular pre-mRNAs (including IFN-β mRNA) through binding to CPSF30.	96, 103, 106, 144, 184-188	(Klemm et al., 2018; Zhu et al., 2022)
DDX21	Cellular	- Regulates the synthesis of vRNAs by inhibiting cellular factors (DDX21, a cellular helicase) that impair viral polymerase activity.	ND	(Chen et al., 2014; Klemm et al., 2018)
DDX56	Cellular	- Human DDX56 protein, an ATP-dependent RNA helicase, classified into the DEAD-box (Asp-Glu-Ala-Asp) protein family- Interacts with IAV NS1 in both yeast and mammalian cells and has a positive regulatory effect on viral replication.	ND	(Pirinçal and Turan, 2021)
DDX3	Cellular	- DDX3, a DEAD-box RNA helicase, interacts with IAV NS1 and NP proteins and exerts antiviral function via impairing the stress granule formation	ND	(Thulasi Raman et al., 2016)
PABPII	Cellular	- Prevents the polyadenylation processing of cellular mRNAs, preventing nuclear export by interacting with PABPII.	223-230	(Chen et al., 1999; Klemm et al., 2018)
Importin-α	Cellular	- Induces IAV NS1 accumulation in the nuclei of infected host cells via binding to importin-α by the NLS1 and NLS2 domains in IAV NS1.	35, 38, 41, 219, 220, 224, 229, 231, 232	(Cheng et al., 2009; Klemm et al., 2018)
hStaufen, eIF4GI and PABPI	Cellular	- Directly activates viral mRNA translation via recruiting cellular host proteins involved in mRNA transport and translation, including hStaufen, the elongation initiation factor 4GI (eIF4GI) and poly-A-binding protein 1 (PABPI) to the conserved 5′ UTR of viral mRNA (AGC(A/G)AAAG).	1-81 and 73-230	(Klemm et al., 2018; Kim et al., 2021)
NXF1/NXT1	Cellular	- Interacts with NXF1/NXT1 to inhibit host mRNA translation.	ND	(Ehrhardt et al., 2007; Klemm et al., 2018)
PDZ	Cellular	- NS1 Interacts with PDZ domain-containing proteins to modulate viral replication by using the PDZ-binding motif at the CTD.	227-230	(Thomas et al., 2011; Klemm et al., 2018)
ADAR1	Cellular	- Binds to the proviral ADAR1 protein and enhance its RNA editing activity to provide optimal viral protein synthesis and replication.	1-73	(de Chassey et al., 2013; Klemm et al., 2018)
TRIM25	Cellular	- Targets the ubiquitin ligase TRIM25 to evade virus recognition by RIG-I and block TRIM25-mediated innate immune responses.	38, 41, 96-97	(Gack et al., 2009; Klemm et al., 2018)
CRK(L)	Cellular	- Interacts with signaling adaptor proteins CRK and CRK-like [CRK(L)] to activate PI3K pathway and inhibit apoptosis.	212-217	(Ehrhardt et al., 2007; Klemm et al., 2018)
PML	Cellular	- Degrades the promyelocytic leukemia (PML) protein, the main component of the ND10 anti-viral complex, by inducing changes in the SUMOylation pattern of the PML nuclear body. This inhibits the formation of the antiviral Daxx-sp100-p53-PML (ND10) complex. - PML degradation also activates the cellular stress with increasing cellular ROS (reactive oxygen species) levels, mitochondrial dysfunction, and p53 accumulation, activating apoptosis of the host cell.	ND	(Das et al., 2024)
Viral Polymerases and NP	Viral	- Interacts with vRNP complexes and regulate its activity through interaction with host negative regulators	1-81 and 123-127	(Shimizu et al., 1994; Robb Nicole et al., 2011)

ND, undetermined.

**Table 2:** Viral and host interactors of SARS-CoV-2 Nsp1.

Target	Type	Mechanism	Interacting motifs	Citation(s)
40S ribosomal subunit	Cellular	- Inhibits innate immune responses by inserting its C-terminal helices into the mRNA entry channel of the ribosome, promoting host mRNA degradation.	CTD KH motif at aa 164-165	(Schubert et al., 2020a)
NXF1–NXT1 protein complex	Cellular	- Inhibit the nuclear export of cellular transcripts by disrupting the interaction between NXF1 and its NPC interactors.	13-129	(Zhang et al., 2021; Tardivat et al., 2023)
JAK/STAT	Cellular	- Interferes with the JAK/STAT signaling pathway to block host antiviral immune responses.	Indirect interaction	(Bouayad, 2020)
eIF3j	Cellular	- Competes with eIF3j for binding to the 40S ribosomal subunit and impairs the ability of eIF3 to bind to the 40S subunit which blocks eIF3 complex in translation initiation.	Indirect interaction	(Lapointe et al., 2021)
IRF3	Cellular	- Blocks the mitochondrial antiviral-signaling protein (MAVS)-induced IFN production by blocking IRF3 phosphorylation.	Indirect interaction	(Xia et al., 2020)
5’-UTR stem loop 1 (SL1)	Viral	- Mediates host mRNAs translation inhibition via binding to the mRNA entry channel of the ribosome. Nsp1 interaction with the 5’-UTR SL1 of the viral mRNAs allows to switch infected cells translation machinery towards SARS-CoV-2 mRNAs.	10-127	(Yuan et al., 2021; Vora et al., 2022; Tardivat et al., 2023)

## 5 IAV NS1 and SARS-CoV-2 Nsp1 inhibition of innate immunity

### 5.1 IAV NS1-mediated inhibition of innate immune responses

**Figure 5:**
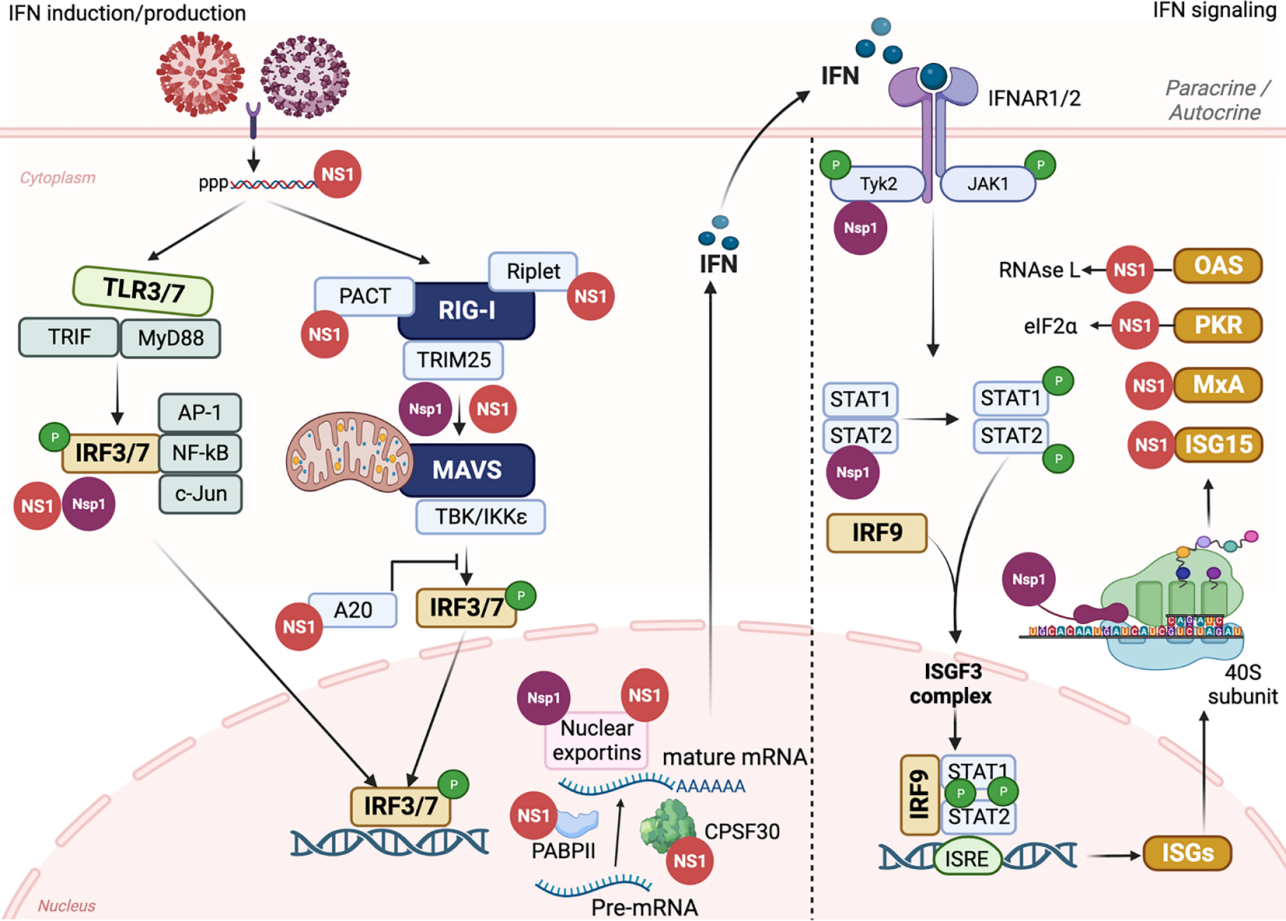
IAV NS1 and SARS-CoV-2 Nsp1 antagonism of innate immune responses. The induction of innate immune response is initiated by triggering TLRs and RLRs pathways after IAV (red) or SARS-CoV-2 (purple) entrance to susceptible cells. The recognition of PAMPs by PRRs leads to the activation of a signal transduction cascade that involves several modulators, including, among others, TRIF, MyD88, PACT, TRIM25, and Riplet, which leads to the phosphorylation of IRF3/7. Nuclear translocation of phosphorylated IRF3/7 induces the transcription of IFN. Through the Janus kinase (JAK)1 and TYK2 signaling pathway, binding of IFN to the IFNR1/2 receptors leads to the phosphorylation of transcription factors STAT1 and STAT2 which together with IRF9 translocate to the nucleus resulting in the expression of hundreds of ISGs with antiviral activity. Both IAV NS1 (red) and SARS-CoV-2 Nsp1 (purple) can interact with various cellular host factors to inhibit activation of IRF3/7, prevent the maturation and nuclear exportation of cellular mRNA, blocking the loading of cellular mRNA to the ribosomes (Nsp1), or counteracting the antiviral response of different ISGs. This figure was created with Biorender.com.

Several studies have elucidated the mechanisms of IAV NS1-mediated inhibition of host immune response (Alonso-Caplen et al., 1992; Ronni et al., 1997; Chen et al., 1999; Shimizu et al., 1999; Talon et al., 2000a; Wang et al., 2000; Geiss et al., 2002; Noah et al., 2003; Kochs et al., 2007; de Chassey et al., 2013; Nogales et al., 2017c). The mechanism of IAV NS1 to inhibit the host innate immune responses could be categorized in three major strategies: 1) inhibition of IFN production; 2) suppression of host gene expression; and 3) inhibition of ISGs (**Figure 5**).

#### 5.1.1 IAV NS1 inhibition of IFN production

IFNs are a complex group of signaling proteins produced by the host cells to interfere with the replication of invader pathogens and mediate the “antiviral emergency alert” for host cells (**Figure 5**) (Garcia-Sastre et al., 1998). IFNs are classified in type I, II, and III. Type I IFNs (IFN-I) are the most important class during IAV infection and includes IFN-α, IFN-β, IFN-κ, and IFN-ω (Medzhitov and Janeway, 1997). IFN-α/β bind to specific cell surface receptor complex, namely IFN alpha receptors (IFNARs), to subsequently activate different signal transduction pathways, resulting in the stimulation of hundreds of ISGs to control the progression of virus replication and infection (**Figure 5**) (Katze et al., 2002).

IAV NS1 modulates the inhibition of IFN production through variable interventions (Baker et al., 2015; Cheng et al., 2015; Nogales et al., 2017a; Nogales et al., 2017b; Nogales et al., 2017c; Hilimire et al., 2020; Martinez-Sobrido et al., 2020; Chiem et al., 2022; Nogales et al., 2022). However, other IAV proteins, including PA-X (Chiem et al., 2021), PB1-F2 (Varga and Palese, 2011; Varga et al., 2012), or PB2 have also been shown to be involved in inhibition of host antiviral responses during IAV infection (Forero et al., 2015; Yang et al., 2022). Following IAV infection, two major cellular pathways are activated to initiate the antiviral innate immune response: Toll-like receptors (TLRs) and RIG-I-like receptors (RLRs) (Iwasaki and Pillai, 2014) (**Figure 5**). TLRs and RLRs are PRRs that can recognize unique PAMPs, such as short 5′ end triphosphate dsRNA that is formed due to the partial complementary sequences in the 5′ end and 3′ end of IAV RNA or the formation of defective viral genomes (DVGs) due to the trailer copyback of the viral polymerase (Pflug et al., 2014; Bosma et al., 2019; Vignuzzi and Lopez, 2019). Mainly TLRs 3 and 7 that are expressed on cells of the respiratory mucosa are involved in IAV recognition (Le Goffic et al., 2007). The recognition of IAV dsRNA PAMPs by TLR3 and TLR7 results in the activation of a signaling cascade, including the recruitment of TIR domain-containing adaptor inducing IFN (TRIF) and Myeloid Differentiation Factor 88 (MyD88) host intermediators, respectively, and activation of IRF3 and IRF7 (IRF3/7), along with other regulatory factors including NF-κB, AP-1, c-Jun/ATF2 (Lund et al., 2004). In a similar pattern, RLRs, including RIG-I, Melanoma Differentiation Antigen-5 (MDA-5), and RIG-I-like receptor (LGP), located in the cytoplasm of host-infected cells can recognize viral PAMPs which lead to dimerization and exposure of caspase activation and recruitment domain (CARD) of RIG-I (Gack et al., 2007) (**Figure 5**). This results in the activation of cellular intermediators namely TRIM25 and RIPLET ubiquitin ligases resulting in the re-localization of RIG-I to the mitochondria for binding to the MAVS adaptor protein and subsequently triggering different tumor necrosis associated factors (TNF). TNF activates multiple kinases, including TBK1 (TANK-binding kinase 1) and IKKε (inhibitor of NF-κB) that phosphorylate IRF3 and IRF7 transcription factors (De Maeyer and De Maeyer-Guignard, 1998) (**Figure 5**). Activation and dimerization of IRF3 and IRF7 through TLRs or RLRs leads to their nuclear translocation to initiate the transcription of IFNs and other immunoregulatory cytokines (Medzhitov and Janeway, 1997) (**Figure 5**). IFNs secreted from infected cells bind to the IFNAR resulting in activation of JAK1 and TYK2 that phosphorylate STAT1 and STAT2 proteins. Phosphorylated STAT1/STAT2, together with IRF9, constitute the IFN-stimulated gene factor-3 (ISGF3) complex that binds to IFN-sensitive response element (ISRE) promoters resulting in the expression of hundreds of ISGs, including, among others, IFN-induced myxovirus resistance proteins (Mxs), IFN-induced transmembrane (IFITM), PKR, and OAS-RNase L to restrict virus replication (Samuel, 2001; Schneider and Sari, 2014) (**Figure 5**).

IAV NS1 has been shown to be implicated in counteracting the host antiviral innate IFN response at multiple levels (**Figures 4**, **5**) (Wang et al., 2000; Geiss et al., 2002; Quinlivan et al., 2005; Nogales et al., 2022). A direct interaction of IAV NS1 to the CARD domain of RIG-I abrogates the activation of all downstream regulatory transcription factors, including TRIM25 and RIPLET ubiquitin ligases that are essential for the priming of IFN production (Bharadwaj and Mohana Priya, 2015) (**Figures 4A**, **5**). Additionally, IAV NS1 can effectively inhibit the activation of RIG-I through direct binding and inhibition of TRIM25 oligomerization (Krug, 2015), and RIPLET (Rajsbaum et al., 2012) ubiquitin ligases (**Figure 5**). It has been shown that aa residues E96 and E97 are important for the binding of IAV NS1 to TRIM25 but not RIPLET (Gack et al., 2009). Recently, a strain specific interaction between IAV NS1 and the second caspase activation and recruitment domain (CARD) of RIG-I (RIG-I 2CARD) was reported to regulate the inhibition of IFN production independent of TRIM-25 pathway (Jureka et al., 2020)

Also, IAV NS1 can inhibit the activation of IRF3 through the induction of ubiquitin-editing protein A20 which downregulates the activation of IRF3 (Feng et al., 2017) (**Figure 5**). Likewise, IAV NS1 can induce the expression of monocyte chemotactic protein-induced protein 1 (MCPIP1) which suppresses cellular inflammatory responses through degrading RIG-I mRNA via its RNase activity (Sun et al., 2018). Inhibition of IFN production through inhibition of IRF3 activation was shown to be variable among different IAV strains (Kochs et al., 2007).

#### 5.1.2 IAV NS1 suppression of host gene expression

In addition to counteracting the activation of IFN production, IAV NS1 can also target host cellular genes and inhibit their expression (Chen et al., 1999; Wang et al., 2002; Noah et al., 2003). Cellular pre-mRNA processing machinery includes the cleavage of the pre-mRNA by the CPSF30 domain at the AAUAAA poly(A) signal followed by poly(A) tail addition to the cleaved product by the help of poly(A) polymerase (PAP) and further elongation by poly(A) binding protein (PABII) (Bienroth et al., 1993) (**Figure 5**). It was shown that CPSF30 also supports PABII to tether the PAP to the RNA template for the elongation (Wahle, 1995) (**Figure 5**). Also, PABII has been shown to play an important role in mRNA export from the nucleus to the cytoplasm (Wahle, 1995). IAV NS1 can bind to CPSF30 and thus induce inhibition of the processes associated with cellular pre-mRNA processing and subsequent antiviral protein expression (Noah et al., 2003; Kochs et al., 2007) (**Figure 5**). The physical association to CPSF30 has been shown to be dependent on particular aa residues in IAV NS1 (**Figure 4A**) (Nemeroff et al., 1998; Kochs et al., 2007). For instance, influenza A/A/PR/8/34(H1N1) (PR8) virus expressing NS1 protein with G184 lacks CPSF30 binding and induces unexpected strong attenuation *in vivo* (Steidle et al., 2010). This lack in the ability of PR8 NS1 to bind CPSF30 was associated with increased IFN production and PR8 attenuation in mice (Bergant et al., 2023). Next to position 184, the position E186 in IAV NS1 ED has been shown to play a role in the interaction with CPSF30 (Noah et al., 2003), including canine IAV (CIV) and equine IAV (EIV) NS1 proteins (Quinlivan et al., 2005; Nogales et al., 2017a; Chauche et al., 2018) (**Figure 4A**).

Additional aa residues, including F103 and M106, have also been shown to be involved in the interaction of IAV NS1 with CPSF30 (**Figure 4**) (Kochs et al., 2007). Because of the differences in the aa sequence of IAV NS1, its binding affinity to CPSF30 is variable between different IAV strains (Kochs et al., 2007; Clark et al., 2017). Notably, two (F103X and M106Y) and six (E55K, L90I, I123V, E125D, K131E, and N205S) aa substitutions were able to restore the ability of NS1 from PR8 and H1N1pdm09 viruses, respectively, to bind to CPSF30 and, therefore, their ability to inhibit nuclear export of cellular mRNAs (Clark et al., 2017).

IAV NS1 also affects the cellular mRNA export machinery by inhibiting several nuclear exportins (NXF1/TAP, p15/NXT, Rae1/mrnp41, and E1B-AP5) that are critical for shuttling mRNA from the nucleus to the cytoplasm of infected cells (Falcon et al., 2004; Satterly et al., 2007) (**Figures 4A**, **5**). As a result of inhibiting the nuclear exportins and the direct inhibition of PABII by IAV NS1, the accumulated cellular mRNA in the nucleus of infected cells will be accessible to the virus PA and PB2 polymerase subunits to snatch the capped-RNA primers which are needed for the transcription of viral mRNA (Falcon et al., 2004). It is noteworthy that viral mRNA poly(A) tail processing is not relying on the cellular mRNA machinery and rather involves the synthesis of poly(A) tail by the virus polymerase complex that uses U residues in the virion RNA as a template (Nemeroff et al., 1998; Falcon et al., 2004).

#### 5.1.3 IAV NS1 inhibition of ISGs

IFN stimulates the expression of more than 300 ISGs that can counteract virus replication through different mechanism, including direct degradation of vRNA, inhibition of mRNA expression, and induction of cell death (Kochs et al., 2007; Kuo et al., 2016; Cheng et al., 2018).

ISG15 is one of the most important and abundant ISGs upregulated during viral infection (Munnur et al., 2022) (**Figure 5**). ISG15 consists of two ubiquitin-like structural domains (Perng and Lenschow, 2018) that binds to the viral target protein and modifies it in a process called ISGylation via the carboxyl-terminal LRLRGG motif (Potter et al., 1999). ISGylation of IAV NS1 (**Figure 3**) resulted in inhibiting its nuclear translocation by preventing homodimer formation and destroying the interaction with α-importins, therefore, affecting NS1-mediated inhibition of host innate immune responses (Zhao et al., 2010).

PKR is another ISG that when activated either by dsRNA or the cellular activator protein, PACT, mediates inhibition of protein translation, including viral proteins, through the phosphorylation of eIF2a (Garcia et al., 2006; Garcia et al., 2007) (**Figure 5**). The aa residues 123-127 in the RBD of IAV NS1 have been shown to be involved in the direct interaction with the PKR junction region, thus, preventing its activation and downstream actions (**Figure 4B**) (Li et al., 2006a; Min et al., 2007).

OAS-RNase L is an IFN-induced antiviral protein which binds to the dsRNA and trigger the activation of ribonuclease L (RNase L) that degrades viral single-stranded (ss)RNA (Silverman, 2007) (**Figure 5**). The RBD of IAV NS1 can shield the dsRNA produced during viral infection from being detected by RNase L and therefore, prevents its activation and downstream actions (Cooper et al., 2015).

### 5.2 SARS-CoV-2 Nsp1-mediated inhibition of innate immune responses

SARS-CoV-2 Nsp1 is an immune modulator and virulence factor that mimics IAV NS1 functions in the replication cycle of SARS-CoV-2. SARS-CoV-2 Nsp1 is also called the host shutoff factor due to its suppressive action on the expression of host cellular genes, including those responsible for inhibiting viral infection (Schubert et al., 2020a; Lapointe et al., 2021) (**Figures 4B**, **5**). SARS-CoV-2 Nsp1 has been shown to suppress host antiviral immune response by acting on the three previously mentioned strategies described for IAV NS1, including shutting off general host gene expression, and inhibiting the expression of IFNs and related ISGs, including those with antiviral activity (Banerjee et al., 2020; Schubert et al., 2020b; Thoms et al., 2020; Tidu et al., 2020) (**Figure 5**).

#### 5.2.1 SARS-CoV-2 Nsp1 suppression of IFN production

Through global inhibition of cellular gene expression, SARS-CoV-2 Nsp1 inhibits the induction of IFN (Yuan et al., 2021). Briefly, Nsp1 inhibits phosphorylation of TBK1/IKKε and IRF3 modulators, thus prevents their nuclear translocation to induce IFN expression during SARS-CoV-2 infection (Xia et al., 2020; Kumar et al., 2021a) (**Figure 5**).

#### 5.2.2 SARS-CoV-2 Nsp1 inhibition of host gene mRNA expression

Several studies revealed that the CTD of SARS-CoV-2 Nsp1 is responsible for inhibiting cellular gene expression through the interaction with the 40S ribosomal subunit (**Figures 4B**, **5**) (Banerjee et al., 2020; Schubert et al., 2020b; Tidu et al., 2020). Briefly, negatively charged aa residues D152, E155, and E159 in the CTD of SARS-CoV-2 Nsp1 which is self-disordered (Kumar et al., 2021b), bind to the positively charged aa residues R116, R117, R143, K148 of uS3 of the 40S ribosomal subunit head resulting in blocking the mRNA entry channel (Shi et al., 2020). Additionally, aa residues K164 and H165 (KH motif) at the CTD of SARS-CoV-2 Nsp1 interact with U607, G625, and U630 of the helix h18 of the 18S rRNA (Banerjee et al., 2020). In parallel, the hydrophobic aa residues F157, W161, L173, and L177 in the CTD interact with the hydrophobic aa residues V106, I109, P111, T122, F124, V147, and I151 of uS5 of the 40S ribosomal subunit (Yuan et al., 2021; Schubert et al., 2023). These findings explained the physical blocking of SARS-CoV-2 Nsp1 to the ribosomal mRNA entry channel, and thus its ability to inhibit cellular gene expression. Also, aa substitutions Y154A/F157A and R171E/R175E were shown to abolish the ability of SARS-CoV-2 Nsp1 to inhibit host gene expression (**Figure 4B**) (Schubert et al., 2020b).

In addition of blocking the loading of cellular mRNA to the ribosomal machinery, SARS-CoV-2 Nsp1 was shown to have endonuclease activity responsible for cleaving cellular mRNAs, but not viral mRNAs unless in a high concentration of the Nsp1, through using a different cleavage pattern for the cellular and viral mRNA (Tardivat et al., 2023). Cleavage of cellular mRNAs requires binding of SARS-CoV-2 Nsp1 to the ribosome particularly the 6-11 nucleotides downstream to the 5’ cap, whereas for the cleavage of viral mRNAs, which occurs only at high concentrations of Nsp1, is mediated at nucleotide positions 45, 46, and 49 downstream to the m7G cap (Tardivat et al., 2023).

The important question is how SARS-CoV-2 Nsp1 inhibits only cellular but not viral mRNA expression, and the answer is because of the 5’ UTR of the SARS-CoV-2 genome. Several studies revealed that the SL1, in addition to SL2, and SL3 hairpins of the 5’ UTR of the SARS-CoV-2 genome, is critical for mediating up-regulation of vRNAs and to escape Nsp1-mediated repression of viral mRNAs (Bujanic et al., 2022). More specifically, three cytosines at positions 15, 19, and 20 in SL1 have been shown essential for antagonizing SARS-CoV-2 Nsp1’s suppression activity (Bujanic et al., 2022). These results explained why all SARS-CoV-2 sub-genomic RNAs encoded a common 50-nucleotide leader sequence that represents the SL1 region (Kim et al., 2020). Additionally, SARS-CoV-2 Nsp1 has been shown to compete with the cellular transcript factor eIF3J for the binding to the 40S ribosomal subunit resulting in inhibition of cellular gene expression (Yuan et al., 2020). Beside the interaction with the ribosomal machinery to efficiently inhibit host gene expression, SARS-CoV-2 Nsp1 interferes with host mRNA nuclear export through interaction with the components of the NPC, including NXF1, NXT1, Nip358, Nup214, Nup153, and Nup62 leading to the accumulation of host mRNA in the nucleus (Zhang et al., 2021) (**Figure 5**).

#### 5.2.3 SARS-CoV-2 Nsp1 inhibition of ISGs

Inhibition of host gene expression prevents IFN production and all regulating functions, including the induction of antiviral ISGs. As discussed earlier, interaction of IFN with the IFNARs results in the activation of a signaling pathway that leads to the induction of hundreds of ISGs with different antiviral functions (Steel et al., 2009). It was shown that SARS-CoV-2 Nsp1 inhibits expression of TYK2 and STAT2 factors during virus infection, without affecting levels of JAK1 and STAT1 expression, which are critical for the induction of ISGs (Kumar et al., 2021a) (**Figure 5**).

## 6 IAV NS1 and SARS-CoV-2 Nsp1 and their contribution to viral replication and pathogenicity

### 6.1 Contribution of IAV NS1 to viral replication and pathogenicity

IAV NS1 is a key multifunctional factor of viral replication, pathogenicity, and disease progression (Garcia-Sastre et al., 1998; Ayllon and Garcia-Sastre, 2015). The establishment of IAV reverse genetics systems allowed the manipulation of NS1 and subsequently uncovering variable roles of IAV NS1 in viral replication and pathogenesis (Hoffmann et al., 2000; Nogales and Martinez-Sobrido, 2016; Blanco-Lobo et al., 2019). Studying the sequence of aa residues located at the linker and disordered CTD of IAV NS1 revealed high variability among different IAV (**Figure 2**) (Bornholdt and Prasad, 2008). Deletion of five aa residues (80-84) located at the NS1 linker region of H5N1 IAV increased virus virulence in chicken and mice (Long et al., 2008), while several studies revealed the impact of NS1 CTD truncations of different lengths on IAV pathogenicity and infectivity (Seo et al., 2002; Quinlivan et al., 2005; Solorzano et al., 2005; Li et al., 2006b; Chambers et al., 2009; Nogales et al., 2022). Moreover, specific aa residues were reported to be crucial as functional sites to mediate viral transcription upregulation, including R38 and K41 (Zhang et al., 2023).

**Figure 6:**
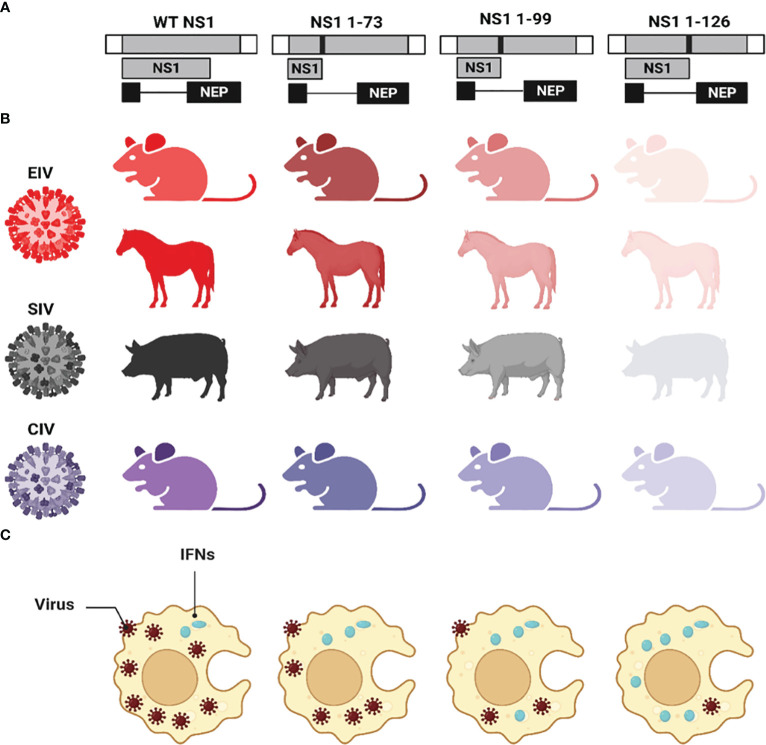
Effect of CTD truncations in IAV NS1 on IAV pathogenicity and virulence. **(A)** Schematic representation of the WT and truncated NS1 1-73, 1-99, and 1-126 of EIV, SIV, and CIV. NS1 and NEP ORF are represented as grey and black boxes, respectively. The 3′ and 5′ NCR of the IAV NS vRNA are represented in white boxes. Black solid lines represent stop codons introduced to generate the NS1 truncations. **(B)** The virulence of each NS1-encoded IAV in different animal species is represented in different colors red (EIV), black (SIV), and violet (CIV). Decreasing the intensity of the color indicates the attenuation of each respective virus. **(C)** The impact of NS1-encoded IAV on the induction of innate IFN responses is represented where IFN induction/production is lowest in case of WT NS1 and increasing proportionally with the length of the NS1 (i.e., NS1 1-73 < NS1 1-99 < NS1 1-126). This figure was created with BioRender.com.

The impact of various NS1 CTD truncations on the replication and pathogenicity of IAV in different animal species have been previously described (Wang et al., 2002; Quinlivan et al., 2005; Solorzano et al., 2005; Chambers et al., 2009; Choi et al., 2015; Nogales et al., 2017b; Huang et al., 2021; Nogales et al., 2022) (**Figure 6**). The expression of only the first 73, 99, or 126 aa residues of the NS1 protein from EIV showed attenuation of virus pathogenicity and virulence compared to the full-length NS1 in mice (Quinlivan et al., 2005) and horses (Chambers et al., 2009) (**Figure 6**). Likewise, the same truncations showed similar pattern of virus attenuation for swine IAV (SIV) (Solorzano et al., 2005) in pigs; CIV in mice and canine tracheal explants (Nogales et al., 2017b) (**Figure 6**); and avian IAV (AIV) in mice and chickens (Steel et al., 2009). Other IAV NS1 C-terminal truncations (NS1 1-86, NS1 1-101, and NS1 1-122) were also reported to induce virus attenuation in mice (Choi et al., 2015). Unlike human IAV where virus attenuation in mice correlated with the length of NS1 [i.e., the shorter NS1 (NS1 1-73), the less virulent the virus] (Talon et al., 2000b; Wang et al., 2002), EIV (Quinlivan et al., 2005; Chambers et al., 2009) and SIV (Solorzano et al., 2005) attenuation did not correlate with NS1 length [i.e., the shortest length NS1 (NS1 1-73) was the most virulent phenotype]. The precise mechanism behind such differences in NS1 truncations among human and other IAV and its correlation to virulence is still unclear, although protein stability, dimerization, specific aa residues, or conformation changes could be responsible for these differences (Wang et al., 2002). Nevertheless, the common hallmark of low virus pathogenicity for all the NS1 C-terminal truncated IAV was attributed to decreasing the ability to antagonize the host innate immune responses which resulted in the early induction of IFN and ISGs. Also, these NS1-truncations revealed the critical role of the CTD in the proper dimerization of IAV NS1 which is critical for the multifunctional roles of NS1 during viral infection (Wang et al., 2002).

Additionally, other individual mutations in IAV NS1 were reported to impact virus pathogenicity: amino acid E139 of NS1 contributed to the high virulence of H5N6 AIV of clade 2.3.4.b in mice (Huang et al., 2021), while aa substitution I64T at the RBD of NS1 from H3N2 human IAV conferred virus attenuation in mice (DeDiego et al., 2016). Also, aa substitutions F103L and M106I at NS1 of H9N2 AIV showed 100% lethality in mice while this lethal phenotype was lost when these two substitutions were reverted to L103 and I106 (Dankar et al., 2011). For SIV, the EPEV and GSEI motifs in the CTD of NS1 have been shown to increase viral replication and virulence in mice by interacting with the cellular PDZ domain that has a critical role is cell signaling, cellular polarity, and act as a scaffold to assemble protein complexes (**Figure 4A**) (Harris and Lim, 2001; Wang et al., 2012). The mouse adaptive F9Y substitution increased the virulence of WSN virus in mice as well as replication efficiency *in vivo* and *in vitro* via enhancing the IFN antagonism properties (Yu et al., 2022). In contrast, the mouse adapted D2I substitution in WSN attenuated the replication and virulence of WSN *in vitro* and *in vivo* via decreasing the IFN antagonistic ability, compared to the NS1-WT protein of WSN (Yu et al., 2022). These and other findings highlight the critical role of NS1 protein in mediating IAV replication, pathogenicity, and virulence.

### 6.2 SARS-CoV-2 Nsp1 contribution to replication and pathogenicity

SARS-CoV-2 Nsp1 is a major virulence factor during virus replication (Frolov et al., 2023). Due to its critical importance in suppressing host gene expression, SARS-CoV-2 Nsp1 hijacks the cellular machinery to promote viral replication and pathogenicity (Frolov et al., 2023). Thus, mutations in SARS-CoV-2 Nsp1 affecting its ability to inhibit host gene expression attenuate virus replication and virulence. For instance, deletion of aa residues at positions 155-165 of SARS-CoV-2 Nsp1 resulted in lower virus replication in Calu-3 cells, and reduced pathogenesis and viral replication in golden Syrian hamsters compared to SARS-CoV-2 expressing a WT Nsp1, demonstrating the contribution of SARS-CoV-2 Nsp1 in viral replication and pathogenesis (**Figure 4B**) (Fisher et al., 2022).

## 7 IAV NS1 and SARS-CoV-2 Nsp1 as targets for antiviral research

Although several drugs are approved for the treatment of IAV or SARS-CoV-2 infections, drug-resistant viruses to currently approved antivirals can emerge, making a serious challenge to treat and control viral infections (Vegivinti et al., 2022; Vitiello, 2022; Li et al., 2023; Moghadasi et al., 2023; von Delft et al., 2023). Therefore, there is an urgent need to find novel and potent antivirals for the treatment of IAV and SARS-CoV-2 infections in humans. A promising strategy to identify and develop compounds with antiviral activity is the pursuit of new viral targets with key functions in the biology of the virus. In this regard, IAV NS1 or SARS-CoV-2 Nsp1, which are required for efficient virus replication, represent promising targets for the identification of inhibitors for the treatment of these viral infections. Moreover, these inhibitors could also be used to increase our current understanding of the key roles of these viral proteins during infection, including virus-host interactions and mechanism(s) of viral pathogenesis (Kim et al., 2021). Here, we briefly describe the advances in the identification of new compounds targeting IAV NS1 (**Table 3**) or SARS-CoV-2 Nsp1 (**Table 4**), which support a model in where inhibition of NS1/Nsp1 functions results in restoration of the host innate immune responses and therefore inhibition of IAV/SARS-CoV-2 replication, respectively.

**Table 3:** Compounds with antiviral activity against IAV NS1.

Compound	Function	Citation(s)
NSC125044	Inhibition of IFN-related NS1 functions.	(Basu et al., 2009)
A9 (JJ3297) and A22 (synthesized based on structural features of NSC125044).	Inhibition of NS1-CPSF30 interaction and likely other IFN-related NS1 functions.	(Walkiewicz et al., 2011; Jablonski et al., 2012; Kleinpeter et al., 2018)
35 and 44	Disruption of dsRNA-NS1 interaction.	(You et al., 2011)
Epigallocatechin gallate (EGCG)	Interaction with the RBD of NS1.	(Cho et al., 2012)
32056 and 31674	Inhibition of NS1-CPSF30 interaction.	(Ai et al., 2014)
Baicalin	Binding to RBD.	(Nayak et al., 2014)
Pyrazolopyridine derivates (compound 32)	Blocking the anti-IFN function of IAV NS1.	(Patnaik et al., 2019)
Pterostilbene.	Inhibition of ubiquitination mediated degradation of RIG-I.	(Wu et al., 2023)
Compounds 157 and 16.	Most likely inhibition of NS1-CPSF30 interaction.	(Marsili et al., 2023)

**Table 4:** Compounds with potential antiviral activity against SARS-CoV-2 Nsp1.

Compound	Function	Ref
**Ganirelix**	Disrupt the NXF1/NXT1-Nsp1 complex	(Vasudevan and Baraniuk, 2021)
**CHEMBL1096281 CHEMBL2022920 CHEMBL175656**	Binding to Nsp1	(Chowdhury and Bagchi, 2022)
**Montelukast sodium hydrate**	Binding to CTD of Nsp1	(Afsar et al., 2022)
**Golvatinib, Gliquidone, and Dihydroergotamine**	Binding to Nsp1	(Shen et al., 2023)
**Glycyrrhizic acid, lobaric acid, garcinolic acid, and tirilazad**	Prevent binding of Nsp1 to SL1 region	(Vankadari et al., 2020)

### 7.1 IAV NS1 as a target for the development of antivirals

In a study to identify compounds that phenotypically suppress IAV NS1 function, a yeast-based assay was used to identify 4 promising candidates (NSC109834, NSC128164, NSC95676, and NSC125044) with antiviral activity against IAV (Basu et al., 2009). Three of these compounds (NSC109834, NSC128164, and NSC95676) reduced viral M2 protein and vRNA levels (Basu et al., 2009). However, despite NSC125044 significantly increasing IFN-β mRNA levels, it did not affect vRNA levels (Basu et al., 2009). Authors demonstrated that IAV NS1 was required for the function of NSC125044, suggesting that IAV NS1 was the direct target of NSC125044 (Basu et al., 2009). Later, a series of compounds based on NSC125044 were designed and evaluated for their anti-IAV activity (Jablonski et al., 2012), showing that compounds A9 (or JJ3297) and A22 (developed based on A9/JJ3297) were more effective in blocking IAV replication (Jablonski et al., 2012). A9 was observed to inhibit IAV replication *in vitro* by several orders of magnitude without cellular toxicity (Walkiewicz et al., 2011). Authors demonstrated that compound A9 did not inhibit IAV NS1 expression or its cellular localization in infected cell (Walkiewicz et al., 2011). However, this antiviral agent facilitated the induction of an IFN-like antiviral state and required the function of RNase L, showing that a competent IFN system is necessary for the antiviral activity of A9 (Walkiewicz et al., 2011). Moreover, authors observed that A9 did not target NS1-dsRNA interaction (Walkiewicz et al., 2011). Later structural analyses revealed the potential molecular mechanism of inhibition of A9 and A22 on IAV NS1 (Kleinpeter et al., 2018). Authors determined that A9 and A22 interacted with the NS1 ED in the hydrophobic pocket known to facilitate binding to the host factor CPSF30 suggesting that both compounds likely attenuate IAV replication by inhibiting NS1 interaction with CPSF30 (**Figure 4A**) (Kleinpeter et al., 2018). In another study, a library of 46 quinoxaline derivatives to target IAV NS1 protein demonstrate that these compounds disrupted the ability of IAV NS1 to interact with dsRNA (You et al., 2011). Compounds 35 and 44 were the most promising candidates, with compound 44 being able to inhibit viral growth ~10-fold (You et al., 2011).

A fluorescence polarization (FP)–based binding high-throughput screening (HTS) assay was also developed for the identification of small molecules blocking dsRNA binding to IAV NS1 protein (Cho et al., 2012). Using a NIH library of compounds, six molecules were identified as possible ligands in an initial screening and epigallocatechin gallate (EGCG) was confirmed to be active against IAV (Cho et al., 2012). Moreover, a mechanism of action for EGCG inhibition of IAV was proposed, involving the interaction with the dsRNA-binding motif of IAV NS1, with aa residue R38 being essential for that interaction (**Figure 4A**) (Cho et al., 2012).

In a separate HTS assay, 30,000 compounds from a Traditional Chinese Medicine database were tested for their ability to inhibit the block of gene expression through the interaction of IAV NS1 with CPSF30 (**Figure 4A**) (Ai et al., 2014). Four compounds were identified as potential inhibitors for IAV NS1, with the drugs 32056 and 31674 as the most promising candidates (Ai et al., 2014). Molecular mechanistic studies suggested that 30256 and 31674 stably bind to the CPSF30-binding site in IAV NS1 with high binding-free energy disrupting the ability of IAV NS1 to interact with CPSF30 (**Figure 4A**) (Ai et al., 2014).

The flavonoid baicalin isolated from the dried root of *Scutellaria baicalensis Georgi*, has been shown to inhibit hepatitis B virus replication (Cheng et al., 2006). In a later study, baicalin was also shown to have antiviral activity against IAV both *in vitro* and in mice, and the molecular mechanism of action was also drawn (Nayak et al., 2014). Authors concluded that baicalin has anti-IAV activity by modulating the function of NS1, resulting in up-regulation of IFN-induced antiviral signaling and a decrease in PI3K/Akt signaling in cells (Nayak et al., 2014). Molecular docking predicted the binding site of baicalin to the RBD of IAV NS1, with aa residues 39-43 being crucial for baicalin-NS1 interaction, disrupting the NS1–p85β interaction, and thus, reducing Akt phosphorylation (**Figure 4A**) (Nayak et al., 2014).

It has been reported the discovery and optimization of novel pyrazolopyridine IAV NS1 protein antagonists, which can potently inhibit IAV replication in cells (Patnaik et al., 2019). Among the 40 pyrazolopyridines tested for their ability to inhibit IAV replication, compound 32 had a potent antiviral activity (Patnaik et al., 2019). Importantly the inhibitory effect of compound 32 was related to its ability to interact with IAV NS1 as demonstrated using a yeast-based assay (Patnaik et al., 2019). None of the most promising compounds directly affected IAV NS1 protein expression in infected cells, suggesting that the drugs were acting functionally (Patnaik et al., 2019). Although the pharmacokinetics of some compounds was studied in mice, the antiviral activity of these inhibitors *in vivo* was not evaluated (Patnaik et al., 2019).

Pterostilbene, a 3,5-dimethoxy analog of resveratrol, was found to inhibit IAV infection *in vitro* by affecting the late stages of viral replication (Wu et al., 2023). A potential interaction between pterostilbene and IAV NS1 protein was suggested, with the participation of a key residue in the CTD of IAV NS1 (**Figure 4A**), H169, as responsible for the inhibition of IAV infection. The analysis for the molecular mechanism of action also indicated that upon IAV infection, pterostilbene can promote the expression of ISGs induced by IFN (**Figure 4A**) (Wu et al., 2023). Further analysis suggested that pterostilbene could interact with IAV NS1, inhibiting ubiquitination-mediated degradation of RIG-I (**Figure 4A**) (Wu et al., 2023).

Finally, a cell-based screening assay was used to interrogate a small molecule library of compounds to identify those that antagonized IAV NS1 protein (Marsili et al., 2023). Two drugs with anti-NS1 activity, compounds 157 and 164, were identified (Marsili et al., 2023). Interestingly, an analysis of the structural motif of drugs 157 and 164 revealed a high similarity with previously described agents JJ3297 (A9) and A22 described above, suggesting that antiviral agents JJ3297, A22, 157, and 164 may act via similar molecular mechanisms of action (Marsili et al., 2023). Importantly, compounds 157 and 164 were able to inhibit the replication of IAV in cell culture (Marsili et al., 2023).

### 7.2 SARS-CoV-2 Nsp1 as a target for the development of antivirals

As described in previous sections, the multifunctional SARS-CoV-2 Nsp1 interacts directly with the NXF1 and NXT1 proteins of the nuclear pore complex disrupting the nuclear mRNA export machinery and, therefore, inhibiting host gene expression (Zhang et al., 2021). The e-Drug3D database, which contained around 2,000 Food and Drug Administration (FDA)-approved drugs, was used to dock to the structure of the NXF1/NXT1-Nsp1 complex to identify compounds that interfere with the binding of SARS-CoV-2 Nsp1 with NXF1/NXT1 (Vasudevan and Baraniuk, 2021). In this *in silico* study, the top identified hit was ganirelix, which competitively antagonizes the gonadotropin releasing hormone receptor (GNRHR) (Vasudevan and Baraniuk, 2021). However, authors did not carry out *in vitro* experimental tests to confirm the antiviral effect of this compound against SARS-CoV-2 (Vasudevan and Baraniuk, 2021).

In other *in silico* study, authors modeled the three-dimensional structure of SARS-CoV-2 Nsp1 to screen two libraries of compounds, the ChEMBL library (Gaulton et al., 2017) and the NCI ligand library (Shiryaev et al., 2011) to identify potential inhibitors of SARS-CoV-2 Nsp1 (Chowdhury and Bagchi, 2022). From this analysis, authors predicted six ligands from the ChEMBL library (CHEMBL1096281, CHEMBL2386944, CHEMBL1761178, CHEMBL2022920, CHEMBL464486, and CHEMBL175656) and six ligands from the NCI library (NCI_232490, NCI_332061, NCI_301755, NCI_657577, NCI_724037, and NCI_215618), with ligands CHEMBL1096281, CHEMBL2022920, and CHEMBL175656 from the ChEMBL library having the best binding affinity values to SARS-CoV-2 Nsp1. However, authors did not perform functional studies *in vitro* or *in vivo* to demonstrate the antiviral activity of the identified *in silico* binding compounds against SARS-CoV-2.

*In silico*, biophysical, and *in vitro* analysis were employed to identify potential FDA-approved compounds targeting the CTD of SARS-CoV-2 Nsp1 (**Figure 4B**), which bind in the mRNA entry channel of the 40S ribosomal subunit, therefore preventing the synthesis of key proteins involved in innate immune responses (Thoms et al., 2020; Afsar et al., 2022). Authors found that montelukast sodium hydrate binds to SARS-CoV-2 Nsp1 *in vitro*, forming a stable complex with the CTD of the viral protein. In addition, in cell culture, the compound was able to inhibit viral replication, indicating that montelukast sodium hydrate can be used as a molecule to design new antivirals against SARS-CoV-2 (Afsar et al., 2022). Later, it was shown in a retrospective study that patients receiving montelukast experienced significantly fewer events of clinical deterioration of SARS-CoV-2 infection compared with patients not receiving the drug (Khan et al., 2022).

In a separate study, Mitoxantrone dihydrochloride was found to have good affinity for the CTD of SARS-CoV-2 Nsp1 (Kumar et al., 2022). However, no functional studies were carried out to demonstrate the antiviral activity of Mitoxantrone dihydrochloride on SARS-CoV-2 infection (Kumar et al., 2022).

A bioinformatics drug screening based on SARS-CoV-2 Nsp1 structure was performed and around 7,500 compounds from three databases were collected for molecular docking and *in silico* evaluation (Shen et al., 2023). The data suggested that golvatinib, gliquidone, and dihydroergotamine were superior to other compounds in binding conformation and free energy (Shen et al., 2023). These drugs could interfere with SARS-CoV-2 Nsp1 binding to 40S protein, resulting in inhibition of SARS-CoV-2 infection (Shen et al., 2023). However, studies to confirm the antiviral properties of these potential antiviral compounds against SARS-CoV-2 were not conducted in this study (Shen et al., 2023).

Previous studies have implicated the ability of SARS-CoV-2 SL1 in the leader region of the 5′ UTR in protecting viral mRNAs against Nsp1-mediated translation inhibition, and, therefore, the potential of identifying compounds affecting the ability of Nsp1-mediated mRNA translation inhibition to identify new drugs with antiviral activity against SARS-CoV-2 (Kamitani et al., 2009). Using an *in silico* analysis to evaluate binding of high potential inhibitors by docking with Nsp1, four compounds (glycyrrhizic acid, lobaric acid, garcinolic acid, and tirilazad) were found to bind Nsp1 with greater affinity and also were found to structurally impede the physical interaction between Nsp1 and SL1, thus preventing mRNA degradation. However, the effect of these drugs in inhibiting SARS-CoV-2 infection was not directly evaluated in these studies (Vankadari et al., 2020; Tam et al., 2023).

## 8 IAV NS1 and SARS-CoV-2 Nsp1 as targets for vaccine development

Reverse genetics approaches have provided researchers an efficient and powerful platform to introduce specific modifications in the viral genome of IAV (Martinez-Sobrido et al., 2020; Nogales et al., 2022) or SARS-CoV-2 (Ye et al., 2020; Mabrouk et al., 2022) with the goal to generate recombinant attenuated viruses that can be implemented as effective live-attenuated vaccine (LAV) candidates for the treatment of viral infections. In this review, we briefly discuss the potential of key IAV NS1 and SARS-CoV-2 Nsp1 mutants for their potential implementation as LAV for the prevention of IAV and SARS-CoV-2 infections.

### 8.1 IAV NS1 for the development of LAV

**Figure 7:**
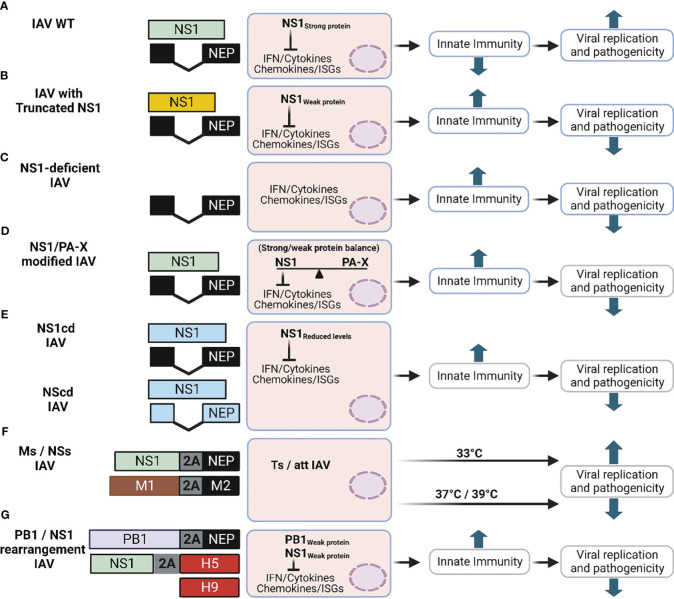
Schematic representation of novel LAV based on IAV NS1. The different approaches described in this review to generate new LAV for IAV based on alterations on NS1 are shown: **(A)** IAV NS WT segment: NS1 and NEP ORF are indicated in light green or black boxes, respectively. For additional information see **Figure 1A**. **(B)** IAV with truncated NS1 (Yellow): Although many truncated IAV NS1 proteins have been described, only one general example is shown. IAV NS segments with truncated NS1 proteins are affected in counteracting host antiviral immune responses (IFN and cytokines), which results in affecting viral replication and propagation. **(C)** NS1 deficient IAV: In this LAV approach, only the ORF of NEP is expressed and the mechanism of attenuation is like the one of viruses encoding truncated NS1 proteins **(B)**. **(D)** IAV with modified NS1 and/or PA-X proteins: In this approach the properties of NS1 and PA-X to inhibit host gene expression are modified, affecting the ability of the virus to counteract innate immune responses. **(E)** IAV with CD NS: IAV expressing codon deoptimized (CD) NS1 (NS1cd) or both NS1 and NEP (NScd) ORF were generated. NS1 CD and NEP CD ORF are indicated with light blue boxes. In this approach reduced levels of NS1 (NS1cd) or both NS1 and NEP (NScd) result in viral attenuation. **(F)** IAV with modified M and NS segments: Recombinant IAV with modified vRNA segment 7 (M, brown) alone or in combination with segment 8 (NS), in which the overlapping ORF of the M1 and M2 proteins (Ms), and NS1 and NEP proteins (NSs) are produced from the same transcript using the PTV-1 2A autoproteolytic cleavage site. Infection with IAV encoding Ms results in viral attenuation at high temperatures (37°C or 39°C), showing a temperature sensitive (ts) and attenuated (att) phenotype. Addition of NSs into the Ms increased the att profile of the modified Ms IAV LAV. **(G)** Rearranged of viral segments 2 (PB1) and 8 (NS): A modified PB1 viral segment encoding PB1 and NEP using the previously described 2A approach results in a reduction in its polymerase activity. The expression of H5 from modified segment 8 containing a truncated NS1 using a FMDV2A autocleavage site results in a bivalent LAV against two different IAV (H9 and H5, red boxes). This figure was created with Biorender.com.

Based on its ability to counteract host antiviral innate immune responses, IAV NS1 can significantly contribute to viral replication and pathogenesis (**Figure 7A**). Many strategies based on partial truncations (**Figure 7B**) or deletion (**Figure 7C**) of IAV NS1 have been used as LAV candidates against IAV infecting humans (Baskin et al., 2007; Wacheck et al., 2010; Pica et al., 2012), swine (Solorzano et al., 2005; Richt et al., 2006; Vincent et al., 2007; Kappes et al., 2012), equine (Quinlivan et al., 2005), canine (Nogales et al., 2017b), and avian (Wang et al., 2008; Steel et al., 2009; Choi et al., 2015; Jang et al., 2016; Jang et al., 2018) hosts [revised in (Nogales et al., 2022)] (**Figure 7**).

Given that IAV NS1 is a virulence factor containing several functional domains or key aa residues involved in different functions, a correlation between the length of IAV NS1 in viral replication and pathogenicity/attenuation has been observed (Nogales et al., 2018b). Although the fitness of NS1-truncated or -deficient IAV is highly affected in IFN-competent systems, these attenuated IAV with deletion/truncations in NS1 are still able to replicate and induce robust humoral and cellular immune responses that protect against challenge with WT IAV in multiple animal species, including birds, mice, pigs, horses, macaques, or humans (Quinlivan et al., 2005; Solorzano et al., 2005; Richt et al., 2006; Baskin et al., 2007; Vincent et al., 2007; Wang et al., 2008; Steel et al., 2009; Wacheck et al., 2010; Kappes et al., 2012; Pica et al., 2012; Choi et al., 2015; Jang et al., 2016; Nogales et al., 2017b; Jang et al., 2018; Nogales et al., 2022). Importantly, the use of NS1-deficient or -truncated IAV could help to develop and implement Differentiating Infected from Vaccinated Animals (DIVA) strategies [revised in (Nogales et al., 2022)].

IAV segment 3 encodes two proteins, PA and the PA-X, which is expressed using a +1 ribosomal shifting mechanism (Jagger et al., 2012). IAV PA-X is also a key virulence factor that together with IAV NS1 counteracts the cellular innate immune response and modulates pathogenicity during IAV infection (Nogales et al., 2018a; Nogales et al., 2018b; Hilimire et al., 2020; Nogales et al., 2021; Chiem et al., 2022). PA and PA-X proteins contain the same first N-terminal 191 aa residues, including the endonuclease domain. However, PA-X encodes a unique C-terminal sequence that contains 41 or 61 aa residues (Jagger et al., 2012). Notably, IAV NS1 and PA-X proteins induce cellular shutoff using different molecular mechanisms (Nogales et al., 2018a; Nogales et al., 2018b; Hilimire et al., 2020; Nogales et al., 2021; Chiem et al., 2022). In addition, PA-X is also involved in modulating inflammatory responses, apoptosis, and cell differentiation (Nogales et al., 2017c; Nogales et al., 2018b). Recent evidences have suggested a functional co-evolution of IAV NS1 and PA-X proteins in order to maintain a strict balance in the inhibition of host gene expression, and this characteristic has been used for the generation of new LAV approaches, using different IAV strains, with different levels of immunogenicity or safety profiles (Nogales et al., 2017c; Nogales et al., 2018a; Hilimire et al., 2020; Nogales et al., 2021) (**Figure 7D**). A set of recombinant H1N1pdm09 encoding PA-X and NS1 proteins with altered abilities to inhibit host gene expression were assessed (Nogales et al., 2017c). All recombinant H1N1pdm09 strains contained the cold adapted (ca), temperature sensitive (ts), and attenuated (att) signature of the Master Donor Virus (MDV) A/Ann Arbor/6/60 H2N2 used for the human LAV (Martinez-Sobrido et al., 2018). Importantly, using a mouse model of infection, lower levels of replication and humoral responses were observed in animals vaccinated with viruses where both viral proteins have, or do not have, the ability to inhibit host gene expression (Nogales et al., 2017c). Likewise, using a similar approach for the MDV A/Ann Arbor/6/60 H2N2 strain, authors demonstrated that removing the ability of PA-X to inhibit host gene expression increased the safety profile of the MDV in mice, while retaining high levels of protective efficacy in the same animal model (Hilimire et al., 2020). These advances could be important to make the human LAV available to individuals currently excluded for the use of this prophylactic option (e.g. immunocompromised or pregnant individuals).

The genetic code is degenerated, because there are 64 codon combinations but only 20 aa (Baker et al., 2015). For that, most aa are coded by different synonymous codons, apart from methionine (M) and tryptophan (W) that are encoded by a single specific codon, AUG and UGG, respectively (Gouy and Gautier, 1982; Athey et al., 2017; Parvathy et al., 2022). Importantly, it has been shown that the DNA/RNA genomes from diverse organisms exhibit favored usage of certain codons over others, and this characteristic is named codon usage bias (Gouy and Gautier, 1982; Quax et al., 2015; Athey et al., 2017). This flexibility of the genetic code has been used for the codon optimization (CO) or codon deoptimization (CD) of genes, increasing or decreasing, respectively, protein expression in different systems. In the last years, CD has been used for viral attenuation and the generation of LAV to prevent infection of different RNA or DNA viruses (Burns et al., 2006; Nogales et al., 2014; Baker et al., 2015; Cheng et al., 2015; Cai et al., 2020; Lorenzo et al., 2022). Taking advantage of current technologies of synthetic biology and established reverse genetics methods, a set of recombinant IAV where the coding regions of both NS1 and NEP, alone or in combination, were CD for the generation of LAV (Nogales et al., 2014) (**Figure 7E**). Viruses harboring the CD NS1 (NS1_CD_) or both CD NS1/NEP (NS_CD_) sequences replicated to high levels in MDCK cells, although viral replication was negatively affected in human lung A549 cells (Nogales et al., 2014). Notably, NS1_CD_ and NS_CD_ IAV were attenuated in a mouse model of infection compared to the WT IAV (Nogales et al., 2014). In addition, a single intranasal immunization dose of IAV NS_CD_ was able to confer protection against subsequent homologous and heterologous IAV lethal challenges, suggesting that this CD approach could be implemented to generate novel LAV candidates for the prevention of IAV infection.

Taking advantage of the segmented genome of IAV, viral genome rearrangement affecting the expression or location of viral genes has been used with promising results for the development of LAV (Nogales et al., 2016). IAV segments 7 (M) and 8 (NS) encode two different proteins (M1 and M2; and NS1 and NEP, respectively) using an alternative splicing mechanism (**Figure 1A**). Using reverse genetics approaches, a set of recombinant IAV with non-splicing NS and/or M segments were generated (Nogales et al., 2016). To that end, the overlapping ORF of the M1/M2 proteins (M segment) and/or the NS1/NEP proteins (NS segment) were separated using the porcine teschovirus 1 (PTV-1) 2A autocleavage site (**Figure 7F**) (Nogales et al., 2016). Notably, mice inoculated with IAV containing the modified M segment, alone or in combination with modified NS segment, were protected against a lethal challenge with WT IAV (Nogales et al., 2016). These viruses were also highly attenuated in the same mouse model of IAV infection (Nogales et al., 2016). The authors demonstrated that viruses encoding the modified M segment were impaired in replication at nonpermissive high temperatures (37°C), whereas high levels of viral replication could be achieved at lower temperatures (33°C) (Nogales et al., 2016).

In another report, a bivalent LAV against H5N1 and H9N2 AIV, both with pandemic potential, was generated by reorganization of the viral genome (Pena et al., 2013; Biggerstaff et al., 2014; Parvin et al., 2018). For that, authors modified the IAV H9N2 PB1 segment to encode the viral NEP separated by the foot-and-mouth disease virus (FMDV) 2A autocleavage site (Pena et al., 2013) (**Figure 7G**). Then, the HA ORF from an IAV H5N1 was cloned downstream of a truncated NS1 (1-99) and separated using another FMDV 2A autocleavage site, allowing for individual expression of both viral proteins (Pena et al., 2013) (**Figure 7G**). Notably, the rearranged H9N2 IAV expressing H5 was attenuated and able to induce effective protection against challenge with H9N2 and H5N1 IAV in mice and ferrets (Pena et al., 2013). Although in this review we focus on strategies affecting the rearrangement of NS segment, many other viral segments have also been reorganized to develop LAV to protect against IAV (Martinez-Sobrido et al., 2020).

### 8.2 SARS-CoV-2 Nsp1 for the development of LAV

**Figure 8:**
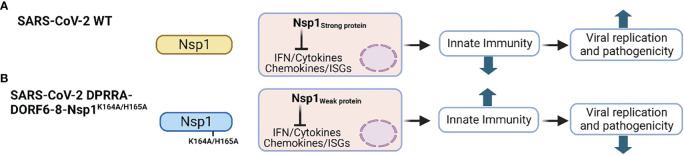
Schematic representation of a LAV approach for SARS-CoV-2. **(A)** Representation of SARS-CoV-2 encoding Nsp1 WT (light yellow). **(B)** In the SARS-CoV-2 LAV approach, the polybasic sequence of the viral S protein and the accessory ORF6-8 proteins were removed, together with K164A and H165A mutations in Nsp1 (light blue) to reduce its ability to inhibit host gene expression. This figure was created with Biorender.com.

While many reports have demonstrated the potential of IAV NS1 mutants as safe, immunogenic, and protective LAV to prevent disease in humans or animals, only a few reports in the literature have shown the potential of SARS-CoV-2 with altered Nsp1 for the generation of protective LAV. Given that SARS-CoV-2 Nsp1 shares some of the functions of IAV NS1 as a virulence factor (**Figure 8A**), it also represents a good target for the development of safe, immunogenic, and protective LAV. A bioinformatics study to analyze the structural and functional features of SARS-CoV-2 Nsp1 for the design of new LAV candidates revealed that the aa sequence of Nsp1 is highly conserved among different SARS-CoV-2 strains (>97.6% homology from 47,427 sequences) and similar to the Nsp1 of SARS-CoV, RaTG13, and pangolin-CoV (84.4, 96.7, and 95.6% homology, respectively) (Min et al., 2020). This comparative study could help to rationale design novel LAV against SARS-CoV-2 based on mutations in Nsp1 or even applied some of the previously described strategies used for SARS-CoV to generate SARS-CoV-2 LAV (Jimenez-Guardeno et al., 2015). A recent report used bioinformatic and biochemical approaches to demonstrate that K164 is a critical aa residue for SARS-CoV-2 Nsp1 inhibition of host gene expression, and other functions (Shen et al., 2021). Therefore, this aa residue could be targeted for the generation of LAV against SARS-CoV-2. Recently, a study described a LAV prototype based on the introduction of multiple modifications in the viral genome, including changes in the SARS-CoV-2 Nsp1 (Liu et al., 2022b). For that, authors removed the accessory ORF6-8 proteins and the polybasic sequence within the S protein and introduced the aa changes K164A and H165A to abolish Nsp1’s ability to inhibit host gene expression (Liu et al., 2022b). This recombinant SARS-CoV-2 (WA1-ΔPRRA-ΔORF6-8-Nsp1^K164A/H165A^) (**Figure 8B**) replicated 100-1,000-fold worse than WT SARS-CoV-2 and induced less lung pathology in two animal rodent models of SARS-CoV-2 infection, K18 human ACE2 (hACE2) transgenic mice and golden Syrian hamsters (Liu et al., 2022b). Notably, vaccination of golden Syrian hamsters with only 100 plaque forming units (PFU) of the attenuated WA1-ΔPRRA-ΔORF6-8-Nsp1^K164A/H165A^ induced strong humoral responses and protected against WT SARS-CoV-2-induced weight loss and pneumonia (Liu et al., 2022b). In a more recent study, the authors showed that intranasal inoculation with this attenuated SARS-CoV-2 induced both mucosal and systemic IgA and IgG responses in golden Syrian hamsters (Stauft et al., 2023).

Interestingly, either direct intranasal vaccination or airborne transmission-mediated delivery of WA1-ΔPRRA-ΔORF6-8-Nsp1^K164A/H165A^ protected against heterologous challenge with different SARS-CoV-2 variants of concern (VoC), including Delta, Omicron BA.1, Omicron BA.2.12.1, and Omicron BA.5 (Stauft et al., 2023). Reduced levels of viral replication and lung inflammation were observed in vaccinated animals, demonstrating the feasibility of implementing WA1-ΔPRRA-ΔORF6-8-Nsp1^K164A/H165A^ as a safe, immunogenic, and protective nasal LAV to protect against SARS-CoV-2 infections, including recently circulating VoC (Stauft et al., 2023). This emphasizes the possibility of including modifications in SARS-CoV-2 Nsp1 that impair its ability to inhibit host gene expression for the development of safe, immunogenic, and protective LAV for the prophylactic treatment of SARS-CoV-2 infections, similar to IAV NS1.

Because of the increasing number of novel LAV approaches described during the last years for IAV and SARS-CoV-2, including those based on modifications of IAV NS1 or SARS-CoV-2 Nsp1, and the advantages of LAV over other vaccine approaches, it is expected that more clinical trials will be launched in the future for the implementation of LAV against IAV and SARS-CoV-2 based on modifications/alterations of the viral NS1 and Nsp1 proteins, respectively.

## 9 Conclusions and future perspectives

IAV and SARS-CoV-2 infections represent a serious threat to human public health and global economy. In this review, we discussed the comparative roles of IAV NS1 and SARS-CoV-2 Nsp1 multifunctional proteins during viral infection. Host innate immune systems restrict IAV or SARS-CoV-2 replication and infection. Consequently, to successfully replicate in IFN-competent cells, IAV and SARS-CoV-2 need to control cellular antiviral responses, including the induction and expression of IFN or ISGs, inflammatory cytokine and chemokine responses, and the activities of host proteins that inhibit virus replication. However, inhibition of the innate immune system must be made without negatively affecting the synthesis of viral proteins, vRNA replication and transcription, viral propagation, or expression of host proteins required for efficient viral replication. IAV NS1 and SARS-CoV-2 Nsp1 are key virulence factors that modulate innate immune response and host protein expression. Interestingly, using different molecular mechanisms, these two viral factors have synergistic effects in inhibiting host protein synthesis in infected cells, a function subjected to a fine balance that can determine viral pathogenesis and fitness. However, because of the recent emergence of SARS-CoV-2, there is currently a better understanding of IAV NS1 functions than those of SARS-CoV-2 Nsp1.

Vaccines and antivirals are the most effective strategies to prevent or control, respectively, viral infections and associated disease. Thus, several efforts have been pursued to develop more effective vaccines and antivirals against these important human respiratory viral pathogens. Because of their important role during viral replication, IAV NS1 and SARS-CoV-2 Nsp1 represent excellent targets for the development and implementation of new vaccine and antiviral approaches. Notably, the application of reverse genetics methodologies has provided researchers with the opportunity to generate recombinant IAV or SARS-CoV-2 with attenuated phenotypes, including deletions, truncations, and mutations in NS1 and Nsp1 for their potential use as LAV for the treatment of IAV and SARS-CoV-2, respectively. In this regard, modulating the functions of IAV NS1 and SARS-CoV-2 Nsp1 and their ability to control host antiviral responses induced during viral infection may be explored to generate novel LAV for the efficient protection against these human pathogens. Although much more advances have been done in the development of prophylactic and/or therapeutic treatments based on IAV NS1, current research directions suggests that SARS-CoV-2 Nsp1, like IAV NS1, is also a suitable target for the development of prophylactic and/or therapeutic approaches for the treatment of SARS-CoV-2 infection.

## Author contributions

AK: Formal analysis, Investigation, Software, Validation, Writing – original draft, Writing – review & editing. AN: Data curation, Formal analysis, Investigation, Writing – original draft, Writing – review & editing. LM-S: Conceptualization, Data curation, Formal analysis, Investigation, Software, Supervision, Validation, Writing – original draft, Writing – review & editing. AM: Conceptualization, Data curation, Formal analysis, Software, Validation, Writing – original draft, Writing – review & editing.
